# Investigation of genetic determinants of cognitive change in later life

**DOI:** 10.1038/s41398-023-02726-6

**Published:** 2024-01-18

**Authors:** Liam Mahedy, Emma L. Anderson, Kate Tilling, Zak A. Thornton, Andrew R. Elmore, Sándor Szalma, Arthur Simen, Meredith Culp, Stephen Zicha, Brian T. Harel, George Davey Smith, Erin N. Smith, Lavinia Paternoster

**Affiliations:** 1 MRC Integrative Epidemiology Unit, University of Bristol, Bristol, BS8 2BN UK; 2 Department of Population Health Sciences, Bristol Medical School, University of Bristol, Bristol, BS8 2BN UK; 3 NIHR Bristol Biomedical Research Centre, University Hospitals Bristol and Weston, NHS Foundation Trust and University of Bristol, Bristol, BS8 2BN UK; 4 Takeda Development Center Americas, Inc., San Diego, CA USA; 5 Takeda Development Center Americas, Inc., Cambridge, MA USA

**Keywords:** Genomics, Prognostic markers

## Abstract

Cognitive decline is a major health concern and identification of genes that may serve as drug targets to slow decline is important to adequately support an aging population. Whilst genetic studies of cross-sectional cognition have been carried out, cognitive change is less well-understood. Here, using data from the TOMMORROW trial, we investigate genetic associations with cognitive change in a cognitively normal older cohort. We conducted a genome-wide association study of trajectories of repeated cognitive measures (using generalised estimating equation (GEE) modelling) and tested associations with polygenic risk scores (PRS) of potential risk factors. We identified two genetic variants associated with change in attention domain scores, rs534221751 (*p* = 1 × 10^−8^ with slope 1) and rs34743896 (*p* = 5 × 10^−10^ with slope 2), implicating *NCAM2* and *CRIPT/ATP6V1E2* genes, respectively. We also found evidence for the association between an education PRS and baseline cognition (at >65 years of age), particularly in the language domain. We demonstrate the feasibility of conducting GWAS of cognitive change using GEE modelling and our results suggest that there may be novel genetic associations for cognitive change that have not previously been associated with cross-sectional cognition. We also show the importance of the education PRS on cognition much later in life. These findings warrant further investigation and demonstrate the potential value of using trial data and trajectory modelling to identify genetic variants associated with cognitive change.

## Introduction

Substantial variation exists in individual levels of general cognitive function, and this variation persists across the life course. Evidence suggests that individuals with higher cognitive function are more likely to stay in school for longer and attain higher qualifications during childhood and adolescence [1]; obtain higher-paying jobs [2] and show healthier lifestyle behaviours throughout adulthood [3]. At the opposite end of the life course, they have greater life expectancy, greater quality of life, and fewer comorbidities in old age, such as Alzheimer’s disease (AD) [4]. Thus, understanding both the environmental and genetic drivers of cognitive function is critical for informing potential prevention and treatment strategies.

The genetic drivers of cognitive function have been previously examined, with several genome-wide association studies (GWAS) identifying genetic variants associated with both global [5] and domain-specific cognitive function (e.g. executive functioning [6, 7] processing speed [7], and verbal memory [8]). Most studies to date have examined associations of the genome with global and domain-specific cognitive function cross-sectionally. For example, Davies and colleagues [5] identified 148 genome-wide significant independent loci associated with cross-sectional general cognitive function, in a sample of 300,486 participants aged 16–102 years from three data sources—CHARGE, COGENT, and the UK Biobank.

Although several genes have been implicated in cross-sectionally measured cognitive function, very few studies have examined genetic drivers of change in cognitive function over time [9–11]. It is important to study change because genetic risk factors (and hence potential factors for intervention) may differ from those observed cross-sectionally in the same way that genetic risk factors for the incidence of diseases such as lung cancer (e.g., *CHRNA5* as a risk factor for heaviness of smoking) may differ from genetic drivers of lung cancer progression (e.g., there is little evidence that smoking heaviness affects lung cancer survival). It is also worth noting that most existing studies examining associations between the genome and cognitive trajectories have been conducted in clinical populations (e.g., in those with AD or mild cognitive impairment). Not only does this make the generalisability of findings to the general population challenging, but it can also result in spurious (biased) associations [12, 13].

In this study, we aimed to perform longitudinal GWASs of trajectories of global and domain-specific (i.e., executive function, attention, language, episodic memory, and learning) cognitive function in a sample of 2515 individuals from the TOMMORROW trial, who were aged 65–83 and cognitively healthy at baseline.

## Materials and methods

### Study design

The TOMMORROW study was an interventional trial to delay the onset of mild cognitive impairment (MCI) due to AD, conducted between 2013 and 2018 across 57 clinical sites from the USA, the UK, Australia, Switzerland, and Germany (ClinicalTrials.gov Identifier NCT01931566). It is a phase 3, multicentre, global, double-blind, placebo-controlled, parallel-group clinical trial conducted by Takeda Pharmaceuticals (Deerfield, IL) in partnership with Zinfandel Pharmaceuticals, Inc. (Chapel Hill, NC). The clinical trial has been described previously [14]. See Supplementary Fig. 1 for the TOMMORROW Trial study design and Supplementary Methods for a further description of the TOMMORROW Trial visit details. Briefly, 3465 individuals, aged between 65 and 83 years at the time of the screening visit (mean age 73.4, SD = 5.3) were selected for inclusion. These participants were defined as ‘high’ or ‘low’ risk (according to a combination of age and two established AD risk genotypes: *APOE* ε2/3/4 and *TOMM40* variable length poly-T variant at rs10524523 (“523”), as shown in Supplementary Fig. 1B). Low-risk individuals were assigned to placebo (*n* = 427) and high-risk individuals were randomised to receive pioglitazone treatment (*n* = 1507) or placebo (*n* = 1531). Study visits were conducted every 6 months over a period of ~4 years. The study was terminated in 2018 for not meeting the futility analysis criteria. The trial results are reported elsewhere [15].

Cognitively normal individuals (defined as total score ≥25 on the Mini-Mental State Examination (MMSE) [16] after age and education adjustment at screening) were selected for inclusion. The TOMMORROW Neuropsychological Battery (TNB), containing tests of attention, executive functioning, episodic memory, learning, and language, was also used to confirm normal cognition in potential participants (see Supplementary Table 1 for a description of the specific tests).

### Ethics

The TOMMORROW trial was conducted in accordance with the requirements of the clinical study protocol, in compliance with the ethical principles that have their origin in the Declaration of Helsinki and the ICH Guidelines for GCP, and approval by corresponding regulatory authorities, and the appropriate institutional review boards and independent ethics committees. Participants gave their written informed consent before screening in the study and participants in the genetic analysis gave additional consent to be included in genetic studies. In addition to regular safety surveillance, the safety of participants was evaluated by an independent Data Safety Monitoring Board.

### Inclusion and exclusion criteria

Key inclusion criteria for the TOMMORROW study required that participants were 65–83 years of age at screening, able to physically perform the cognitive tests, and were fluent in the language that tests were administered. They were also required to be cognitively normal at baseline, indicated by the Clinical Dementia Rating Scale (CDR) [17] global score = 0, and by having at least one memory test from the TNB above −1.5 standard deviation (SD) of the demographically corrected normative mean. Participants were also required to have a project partner able to provide information on their cognitive, functional and behavioural status.

Key exclusion criteria included a current diagnosis or history of any type of cognitive impairment or dementia; neurological/psychiatric disorder, significant psychiatric illness, or of any other diagnosis that could significantly affect cognitive performance; alcohol or drug abuse; macular oedema or macular degeneration; congestive heart failure (New York Heart Association Class III–IV); bladder cancer; any cancer in remission for <2 years from screening; hypersensitivity or allergies to pioglitazone or related compounds; postmenopausal fractures with no or minimal trauma; or clinically significant unstable illness. In addition, individuals were excluded from participation if they had been exposed to the cognitive tests performed in this study (with the exception of the MMSE) within 6 months before screening or if they or the study staff participating in this study were aware of the individual’s *TOMM40* or *APOE* status. Participants with unexplained microscopic/macroscopic haematuria or lab values indicating that an individual may have undiagnosed diabetes, liver abnormalities, or positive tests for hepatitis B or C at baseline were also excluded. Any condition or medication that could interfere with the assessments of safety, tolerability, or efficacy, or any concurrent participation in another interventional clinical study was not allowed.

### Clinical assessments

The two types of study visits after baseline were: (1) an in-clinic visit scheduled at 6‐month intervals and (2) a comprehensive medical follow-up visit (CMFV) that occurred usually within 30 days after a participant met protocol-specified trigger criteria at the regular 6-month visit. Cognitive assessments collected at these visits are listed in Supplementary Table 1. Measurement occasions ranged from 1 to 11 occasions. Following a CMFV, participants continued regular 6-month visits unless an investigator decided to withdraw the participant from the trial, or an adjudication decision determined that the participant met the criteria for a primary endpoint. See ref. [18] for a detailed description of the adjudication process of individuals who transfer from MCI to AD.

### Cognitive outcomes

Individuals completed assessments of attention, learning, language, episodic memory, and executive functioning at all measurement occasions (see Supplementary Table 1 for a description of each task). The *z*-scores for the TNB tests at each time point were standardised to the baseline mean and SD. The TNB domain scores for episodic memory, executive function, language, learning, and attention were obtained by averaging selected individual TNB component *z*-scores; the TNB composite score was obtained by averaging the five domain scores. The *z*-scores for the individual domains and the global cognitive function score were used in the analyses.

### Genotyping and imputation

DNA samples were genotyped using Illumina Infinium Omni Express Exome array. All samples achieved a call rate of >99%. Relatedness was calculated using PLINK v1.9 (https://www.cog-genomics.org/plink/) and related subjects (PI_HAT > 0.10) were removed. As the majority of participants were of White/European ancestry, those who reported being of a non-White ancestry or who showed genetic ancestry that was dissimilar to individuals of European ancestry by principal component analysis were excluded. Subjects with genetically estimated sex that did not match their self-reported sex were removed. Sites with a call rate <95% or Hardy–Weinberg equilibrium *p*-value < 1 × 10^−8^ were excluded. This resulted in 2515 subjects and 924,458 variants. Imputation was performed on the 2515 individuals using the reference panel of the NHLBI Trans-Omics for Precision Medicine (TOPmed) project [19] on the TOPMed Imputation Server. A total of 8,784,549 variants with minor allele frequency (MAF ≥ 1%) and imputation *r*^2^ ≥ 0.7 were included in the final analysis.

### Statistical analysis

**Fig. 1:**
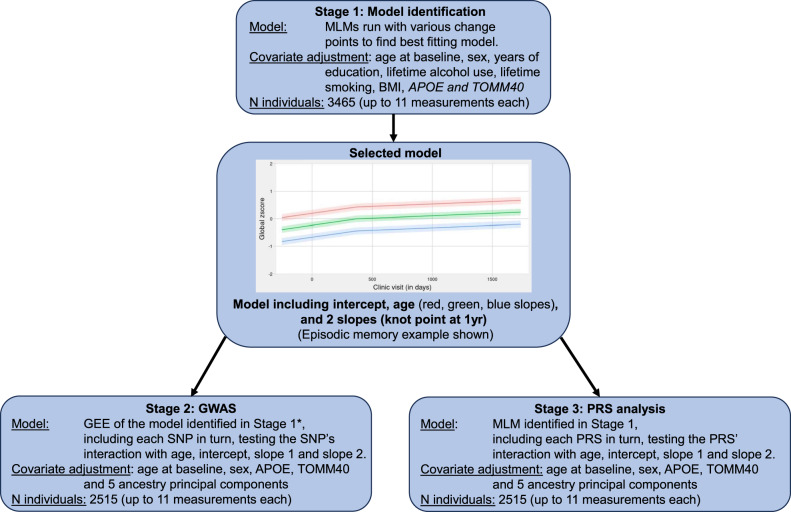
Schematic of the study design. Stage 1 involved model identification of the trajectories and determined that models with a knot (change) point at 1 year adequately represented the data. The resulting models were then used in GWAS and PRS analyses (adjusting only for non-heritable confounders and the genetic factors that affected selection into the trial). *Multilevel models were used for initial model exploration and PRS analysis. For the GWAS, the time taken to fit each MLM meant they could not be used. Instead, we used GEE, which treats the correlation between repeated measures as a nuisance parameter and is therefore much faster to fit. MLM multilevel model, GEE generalised estimating equation, GWAS genome-wide association study, SNP single nucleotide polymorphism, PRS polygenic risk score, BMI body mass index.

A schematic of the analyses is shown in Fig. 1. Multilevel models (MLM) and generalised estimating equations (GEE) were used to account for the correlation between repeated measures on the same subjects. Multilevel models were used for stage 1 model exploration, as these allow estimation of the variability in intercepts and slopes. For the GWAS, the time taken to fit each MLM meant they could not be used. Instead, we used GEE, which treats the correlation between repeated measures as a nuisance parameter and is therefore much faster to fit. For a normally distributed continuous variable, GEE and MLM give comparable estimates—and this was checked. For the PRS modelling MLM was used. Analyses were conducted in R version 4.2 [20, 21]. For both MLM and GEE, models included one knot point at 1 year post-baseline, to allow different linear slopes from baseline to 1 year and from 1 year to the end of the study. Thus, our models estimated average cognitive function (global and domain-specific) at baseline, how average cognitive function changed with age at baseline, and the average change in cognitive function between baseline and 1 year, and between 1 year and the end of the study. Positioning of the knot point was determined using visualisation of average cognitive function scores at each measurement occasion to estimate any slope changes (Stage 1, model identification, Fig. 1) [22].

In stage 1, model identification, the MLM model was adjusted for factors known (from the trial protocol) to be associated with selection into the trial and high/low-risk status (i.e., age at baseline, sex, years of education, lifetime alcohol use, lifetime smoking, BMI, *TOMM40* and *APOE*). We did not adjust for the trial arm because we were including the low-risk (non-randomised) arm and entry into this arm was affected by the selection factors, so including it could have induced selection bias [22]. 3465 individuals completed the baseline assessment, comprising 20,440 rows of individual data. To account for nonindependence within individuals, an unstructured correlation structure (which allows all correlations between timepoints to differ) was used.

We examined factors related to selection by carrying out a GWAS of age and sex among the participants—genetic variants should be independent of both age and sex, but associations could be induced for any genetic variant causing selection into the trial if age and sex cause selection. Any variants shown to be strongly related to age or sex would likely be related to selection into the trial and therefore would be included as covariates in further models [22]. However, only *TOMM40* and *APOE* showed evidence of being associated with selection (see the section “Results”), thus these (together with age and sex) are the only factors that needed to be included to minimise selection bias.

GWAS (Fig. 1, stage 2) were run using GEE models adjusted only for age at baseline, sex, *TOMM40* and *APOE*, and the top 5 ancestry principal components. We did not adjust for years of education, lifetime alcohol use, lifetime smoking, BMI, as these are all likely influenced by (i.e. downstream of) genetic variants, and thus including them could induce selection bias. Six GWAS were run (global cognitive function and the 5 specific cognitive domains), each comprising ~8.8 million individual GEE models (one per variant), with the variants included as additive effects with interactions with age, the intercept, and both slopes. See Supplementary Methods for information on computational processing time. Following analysis, results were additionally filtered for expected minor allele count (EMAC) > 200 (EMAC = 2*N*minor allele frequency (MAF)*imputation quality score (*r*^2^)), which corresponds to a (fairly strict) MAF of 4% where imputation quality is 1 (resulting in 6,498,386 variants remaining). Results were clumped in FUMA [23] to identify independent significant variants using pairwise LD (*r*^2^) of variants in the 1000 genomes Phase 3 European ancestry reference panel [24]. MAGMA gene-based analysis was also run in FUMA [23] and *p* < 3 × 10^-6^ (number of genes tested) was used to identify any additionally associated loci.

In addition to identifying novel associations with GWAS, genome-wide significant associations for cognition function and cognitive change were identified from the following published studies: general cognitive decline [11], attention [9], executive functioning [10], and cross-sectional studies of general cognitive functioning [5], and language [25]. We report the associations observed for these previously identified variants in our corresponding models.

### Sensitivity analyses

To examine the likelihood of bias due to missing genetic data, GEE models were conducted for individuals who completed clinic measures (*n* = 3465) and for the subgroup who had full genotype data (*n* = 2515). Model coefficients were consistent across these two samples (Supplementary Table 2).

### Polygenic risk scores (PRS)

In order to test whether genetic contributions to a set of related traits were associated with cognitive decline, we constructed polygenic risk scores for the related traits; general cognitive ability [5] and AD [26], as well as several traits which have been identified as risk factors for AD or cognition [27]: Alcohol consumption [28], depression [29], education [30], hearing loss [31], low-density lipoprotein (LDL) cholesterol [32], obesity [33], physical activity [34], systolic blood pressure [35], tobacco smoking [36], type 2 diabetes [37], and height [33]. Independent variants with *p* < 5 × 10^−8^ were included, with variants weighted by the effect size from the original GWAS. We tested each PRS for association with all cognitive domains (Fig. 1, stage 3) using multilevel models from the lme4 R package models, adjusting for the same confounders as in the GWAS (age at baseline, sex, *TOMM40* and *APOE* and the top 5 ancestry principal components) and reporting the four parameters (age, intercept, and two slopes) per model.

### *Gene* prioritisation

The Open Targets Genetics platform [38, 39] and FUMA [24] were used to identify the genes with the best evidence of implication at each locus. For the lead variant at a locus, Open Targets creates a score for each gene in a 1 Mb region, which combines evidence from pQTL, sQTL, eQTL, FANTOM5, Promotor capture Hi-C, DNAse hypersensitivity and VEP sources. FUMA reports similar evidence (we investigated positional, eQTL and chromatin interaction gene-mapping evidence, using all available datasets) but for all associated variants in the region. Where FUMA or Open Targets evidence implicated a certain gene through QTL evidence we further investigated this association with colocalization.

### Colocalization

Where eQTL evidence was identified for variants of interest, we further tested these signals for colocalization using pairwise conditional colocalization analysis (PWCoCo) [40]. Colocalization was tested using 1 Mb regions around each gene of interest from the relevant cognition GWAS and the eQTLGen full cis-eQTL dataset (2019-12-11 version) [41].

## Results

### Cognitive trajectories

**Fig. 2:**
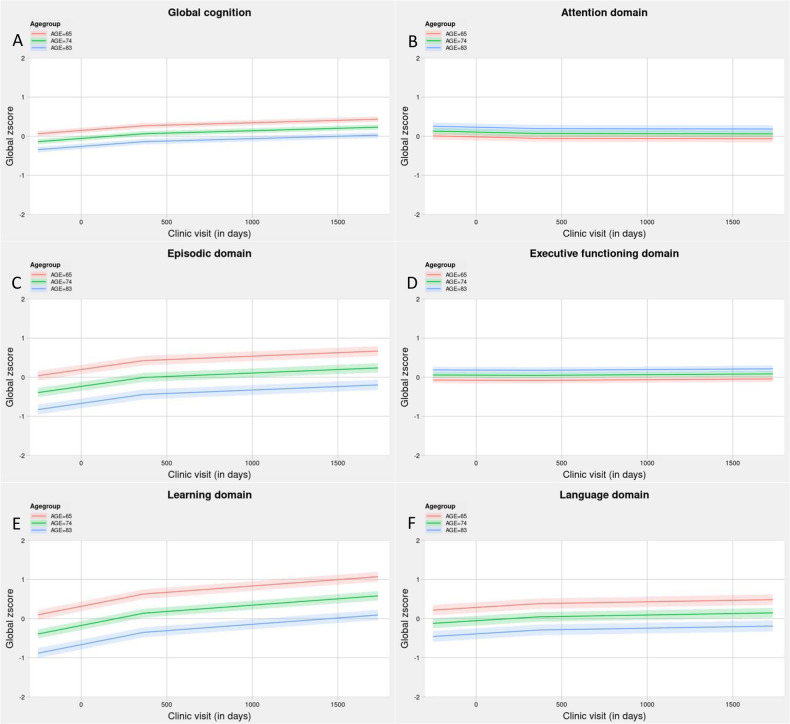
Trajectories of cognitive functioning separated by minimum age at baseline, mean age, and maximum age at baseline in the TOMMORROW Trial for each of the six cognitive domains. **A** Global cognitive functioning, **B** attention domain, **C** episodic memory domain, **D** executive functioning domain, **E** learning domain and **F** language domain. All have a knot point at 365 days, given 2 slopes in the model.

Trajectories of cognitive functioning are presented in Fig. 2. Sample characteristics are presented in Supplementary Table 3. Generally, cognitive performance was lower for people who were older at baseline, but slightly increased over time in the study. The two exceptions to this were for the attention domain and the executive function domain, which showed little change over time during the study, but were slightly higher for people who were older at baseline. Results from the observational multilevel models are presented in Supplementary Table 4. Generally, baseline age (all *p* < 5 × 10^−6^), sex (all *p* < 0.05), and years of education (*p* < 5 × 10^−4^, apart from the attention domain), were associated with the baseline global cognitive function and all specific cognitive domains. There was little evidence for associations between the risk factors/confounders and either slope.

### Selection bias

GWAS of the age of the trial participants showed evidence for selection bias, as demonstrated by a genome-wide significant signal that cannot plausibly be causal (Supplementary Fig. 2a). The bias was limited to a region of chromosome 19 which harbours *APOE* and *TOMM40*, demonstrating that, as expected, only this region of the genome is affected by selection bias and therefore adjustment of *APOE* and *TOMM40* is adequate to mitigate this bias. There was no such obvious bias observed in the GWAS of sex (Supplementary Fig. 2b).

### GWAS

**Fig. 3:**
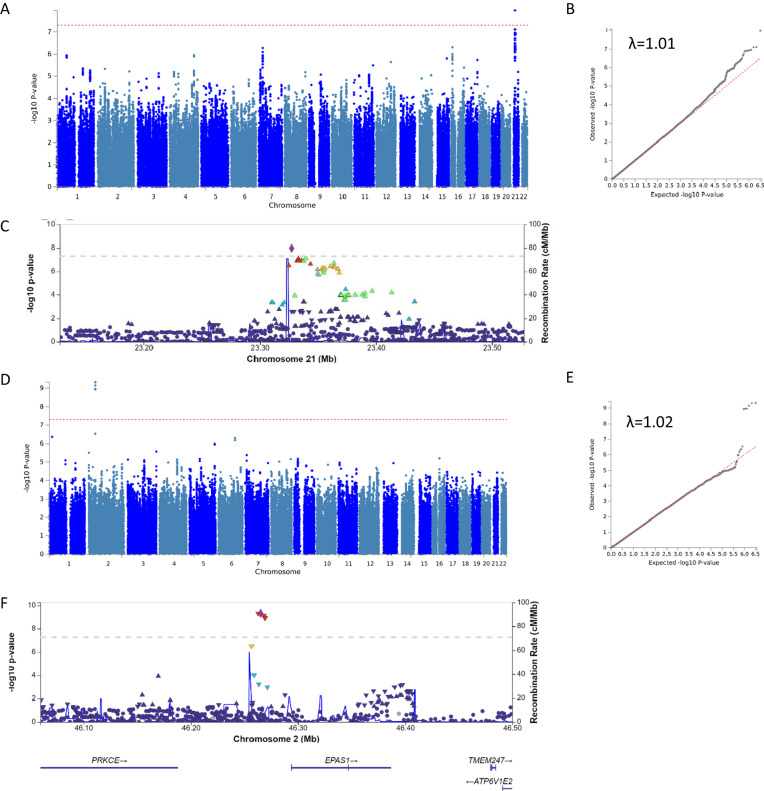
GWAS results for attention slope 1 and slope 2. **A** Manhattan plot of the GWAS of slope 1 in the attention domain. The dotted red line indicates the thresholds for the genome-wide significance of 5 × 10^−8^. **B** QQ-plot **C** GWAS regional Manhattan plot of chromosome 21 for slope 1 in the attention domain. Colours indicate the LD values (*r*^2^) of variants with rs534221751 (in purple). NB—chr21 region is in a gene desert. **D** Manhattan plot of the GWAS of slope 2 in the attention domain. The dotted red line indicates the thresholds for the genome-wide significance of 5 × 10^−8^. **E** QQ-plot **F** GWAS regional Manhattan plot of chromosome 2 for slope 2 in the attention domain. Colours indicate the LD values (*r*^2^) of variants with rs34743896 (in purple).

GWAS QQ plots of the four parameters for the six cognitive measures showed little evidence of genomic inflation (lambda range = 1–1.04, Supplementary Fig. 3). No variants met the genome-wide significance (*p* < 5 × 10^−8^) for any parameter in the GWAS of global cognitive functioning, or for four of the five specific domains (Supplementary Fig. 4). However, in the attention domain GWAS, rs534221751 associated with slope 1 (*p* = 1 × 10^−8^) and rs34743896 associated with slope 2 (*p* = 5 × 10^−10^, Fig. 3, Table 1, both variants imputation quality *r*^2^ = 0.99). These results were substantively unchanged when additionally adjusted for ‘clinical site’ (*p* = 1 × 10^−8^ and *p* = 8 × 10^−10^, respectively). MAGMA gene-based analysis found no additionally associated (*p* < 3 × 10^−6^) loci.

**Table 1:** Lead variants for the genome-wide associated loci in the attention domain.

	Chr: Pos	Effect allele (freq)	Intercept		Baseline age		Slope 1		Slope 2		Prioritised Gene	V2G evidence
rsID			beta	*P*	beta	*P*	beta	*P*	beta	*P*		
rs534221751	21: 23,327,457	A (0.108)	−0.13	0.01	0.01	0.33	**1.30**	**1.1e−08**	−0.22	0.07	–	–
rs34743896	2: 46,264,984	T (0.072)	−0.01	0.73	0.002	0.72	0.54	0.03	**−0.82**	**4.7e−10**	EPAS1	Distance

Chr:Pos are according to build 38. Prioritised genes are the top genes listed by Open Targets Genetics [38, 39] and the evidence used in this assessment are listed in the V2G (variant to the gene) evidence column.

Associations with *p* < 5 × 10^−8^ are marked in bold.

**Fig. 4:**
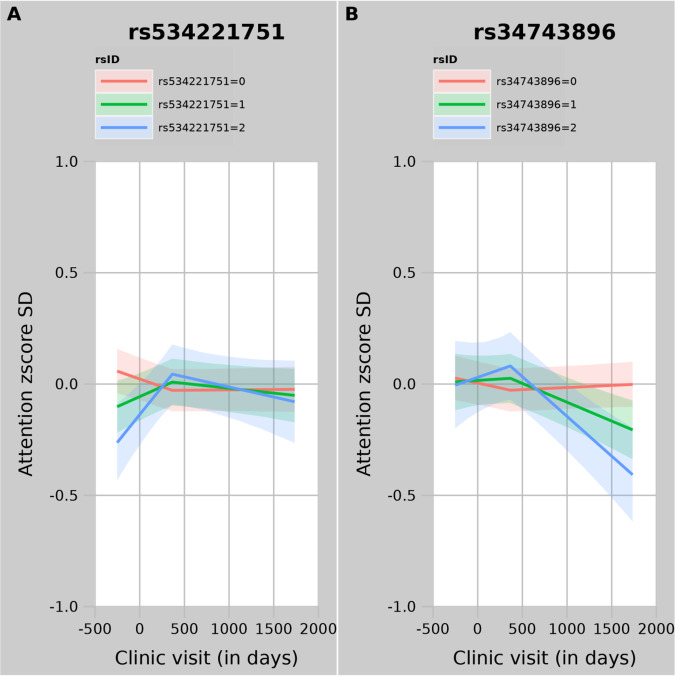
Variant effects on attention domain trajectories. Trajectories plotted correspond to 0, 1 and 2 copies of the minor allele. Panel **A** displays variant rs534221751 for slope 1 in the attention domain; panel **B** displays variant rs34743896 for slope 2 in the attention domain.

Indel rs534221751 showed a weak association with the attention domain score at baseline (*p* = 0.01, with the deletion associated with a lower score), strong evidence for association with slope 1 (*p* = 1 × 10^−8^, with the individuals harbouring 1 or 2 copies of the deletion showing an increase in score and individuals without the deletion showing a decrease in score over the first year of the study) and only weak evidence (*p* = 0.07) for an association (in the opposite direction) with slope 2 (Table 1). This resulted in genotypes showing variation in attention domain scores within the early stages of the trial, but which had converged by the end of the trial (Fig. 4a). In contrast, the T allele of rs34743896, showed little evidence for an effect on attention domain scores at baseline (*p* = 0.73), weak evidence for a positive association with slope 1, but strong evidence (*p* = 5 × 10^−10^) for a negative association with slope 2 (Table 1), resulting in there being little evidence for a difference in attention domain scores by genotype in the early stages of the trial, but a marked divergence towards the end, with individuals with TT genotype showing the greatest decline in attention domain score by year 5 (Fig. 4b).

We examined the associations of these two variants with the other domains. There is some evidence that rs534221751 is also associated with slope 1 of the global cognition model (*p* = 0.0002), but rs34743896 is not associated with slope 2 of any of the other cognitive domains (Supplementary Table 5). These two variants were not associated (i.e., *p* > 1 × 10^−5^) with any other diseases or traits in Phenoscanner.

### Prioritisation of genes at loci

The indel rs534221751 is not available in Open Targets, but proxy variant (rs35743227, *D*’ = 1.0, *r*^2^ = 0.98, 2.4 kb away) showed no prioritised genes. This variant falls within a gene desert on chromosome 21, with the nearest gene, *MRPL39*, >2 Mb away. FUMA (which utilises all variants in the region) also found no significant eQTL evidence but did uncover chromatin interaction evidence between the GWAS locus and *NCAM2* in human embryonic stem cells (FDR = 8 × 10^−11^).

rs34743896 is an intergenic SNP on chromosome 2. The closest gene is *EPAS1* (~28 kb away) but in Open Targets and FUMA, higher prioritisation is given to two more distal genes (*ATP6V1E2*, 277 kb away and *CRIPT*, 351 kb away) based predominantly on eQTL evidence in blood. The variant associated with the steepest decline in attention domain score in our study is associated with lower *ATP6V1E2* expression (*p* = 1 × 10^−22^) and higher *CRIPT* expression (3 × 10^−12^) in blood data from eQTLGen [41]. These two genes also showed chromatin interaction evidence in FUMA in IMR90 mesenchymal stem cells (*CRIPT* FDR = 2 × 10^−16^; *ATP6V1E2* FDR = 6 × 10^−9^). However, there was little evidence that either of the eQTL signals colocalize with the respective GWAS signals (posterior probability for colocalization <0.01% for both). There was no evidence for this region being associated with gene expression in available brain eQTL datasets in Open Targets (lead variant) or FUMA (all associated variants in the region). In the eQTL catalogue [42] (accessed via FIVEx [43]) the strongest association with gene expression in the brain (out of the 139 gene-tissue combinations tested, within 500 kb) was with *PIGF* (*p* = 0.004), which does not meet a Bonferroni correction.

### Exploring previous associations

We also looked up previously reported cognition variants in our GWAS results and found little evidence for association (Table 2 and Supplementary Tables 6–8). Amongst the three variants that had previously shown an association with cognitive change [9–11], only rs11023139 in *SPON1*, previously associated with global cognitive decline in Alzheimer’s disease patients in Sherva et al. [11], met a nominal significance level (*p* < 0.05). This variant showed some evidence for interaction with age at baseline (beta = −0.01, *p* = 0.05) and association with slope 2 (beta = 0.22, *p* = 0.03) in our global cognition model (Table 2), effect allele not reported in the original study.

**Table 2:** Previously identified genome-wide significant variants from GWAS of cognitive decline.

Global cognitive functioning	Sherva et al. [11] (*N* = 303) Alzheimer’s disease assessment scale in AD patients						TOMMORROW Trial (*N* = 2515) Global cognition in cognitively normal individuals			
							Intercept	Age at baseline	Slope 1	Slope 2
	*N*	rsID	Beta (*p*-value)	Effect allele	Population	Phenotypic measures	Beta (*p*-value)	Beta (*p*-value)	Beta (*p*-value)	Beta (*p*-value)
In global cognition	303	rs11023139	0.31 (7.00e−11)	? / A	Clinical—Alzheimer’s disease patients	Alzheimer’s Disease Assessment Scale-cognitive subscale. Memory, language, praxis, attention etc.	0.10 (0.06)	−0.01 (0.05)	−0.22 (0.28)	0.22 (0.03)
**Executive functioning**	**Wendel et al**. [10] **(*N*** = **1338)** **A genome-wide association study of the longitudinal course of executive functions**						**TOMMORROW Trial (*N*** = **2515)** **Global cognition in cognitively normal individuals**			
	***N***	**rsID**	**beta (*p*-value)**	**Effect allele**	**Population**	**Phenotypic measures**	**Beta (*p*-value)**	**Beta (p-value)**	**Beta (*p*-value)**	**Beta (*p*-value)**
In global cognition	1338	rs150547358	1.16 (7.20e−10)	C/-	Clinical - affective-to-psychotic spectrum; and controls	Set-shifting (Trail-Making Test, part B (TMT-B)) and updating (Verbal Digit Span backwards)	–	–	–	–
In EF domain	1338	rs150547358	1.16 (7.20e−10)	C / C			0.67 (0.38)	−0.01 (0.44)	0.21 (0.75)	−0.43 (0.07)
**Attention domain**	**Kamboh et al**. [9]**, (*N*** = **1145)** **Population-based genome-wide association study of cognitive decline in older adults free of dementia: Identification of a novel locus for the attention domain**						**TOMMORROW Trial (*N*** = **2515)** **Global cognition in cognitively normal individuals**			
	***N***	**rsID**	**Beta (*p*-value)**	**Effect allele**	**Population**	**Phenotypic measures**	**Beta (*p*-value)**	**Beta (*p*-value)**	**Beta (*p*-value)**	**Beta (*p*-value)**
In global cognition	1145	rs9535753	0.28 (3.37e−08)	C/C	General	Trail-making Test A	−0.04 (0.13)	0.01 (0.06)	0.12 (0.26)	−0.02 (0.72)
In attention domain	1145	rs9535753	0.28 (3.37e−08)	C/C	General	Trail-making Test A	0.14 (0.59)	−0.002 (0.62)	−0.16 (0.41)	−0.06 (0.56)

Amongst the larger list of variants that have been associated with cognition in cross-sectional studies, there was also little evidence for association in our analyses. Only 6 of the 166 Davies variants had a *p* < 0.01 in our global cognition analysis (the amount you would expect by chance accounting for 166 variants and 4 parameters) and none reached a Bonferroni corrected *p*-value threshold (*p* < 8 × 10^−5^) (Supplementary Table 6). Only 2 of the 127 variants in the Hatoum et al. [6] study had *p* < 0.01 in our attention domain analysis (fewer than you would expect by chance), and none reached a Bonferroni corrected *p*-value threshold (*p* < 1 × 10^−4^) (Supplementary Table 7). None of the three variants associated with MRI-based measure language functional connectivity [25] were associated with our language domain analysis (Supplementary Table 8).

### Association of cognition trajectories with PRS of related traits

Despite the fact that there was little evidence for association amongst the individual 166 variants identified by Davies et al. [5], overall the cognition PRS showed evidence (*p* < 0.008, accounting for 6 models) for an association with baseline in the expected direction for global cognitive functioning and attention, learning and language domains (*p*-values ranged from 0.006 to 5 × 10^−5^, Supplementary Table 9, Supplementary Fig. 5). However, the association with episodic memory was much weaker (*p* = 0.06) and there was little evidence for an association with executive functioning (*p* = 0.280), although estimates for all domains were in the expected direction. There was also some weaker evidence that this PRS was associated with change for some of the domains (e.g., *p* = 0.002 for slope 1 of the attention domain, Supplementary Table 9, Supplementary Fig. 5).

There was no evidence (*p* > 7 × 10^−4^, accounting for 72 models) for association between the PRS for Alzheimer’s disease, obesity, height, tobacco smoking, alcohol consumption, depression, hearing loss, systolic blood pressure, physical activity, LDL, diabetes and any of the parameters across all cognitive domains (Supplementary Table 10, Supplementary Fig. 5). However, the educational attainment PRS [30] showed evidence for association with baseline global cognition (*p* = 5 × 10^−6^) and suggestive evidence (p < 0.05) with all specific domains, other than executive functioning (Supplementary Table 10, Supplementary Figs. 5, 6). The strongest association was seen with the language domain (*p* < 2 × 10^−16^). The attention and language domains also showed some suggestive evidence for an interaction between the education PRS and age at baseline, with the genetic effect getting weaker with age (attention domain: beta = −0.005, *p* = 0.01; language domain: beta = −0.006, *p* = 0.05).

## Discussion

In our genetic analysis of cognitive change, we find preliminary evidence for the involvement of two novel loci with short-term change in the attention domain score in a cohort of individuals aged 65 or over who were cognitively normal at baseline. We find only weak evidence that a small number of variants previously associated with cognition (or change in cognition) are associated with baseline cognition or change in our study, although a PRS combining cognition variants were strongly associated. We also find strong evidence that the PRS for educational attainment is associated with baseline cognition amongst individuals aged 65 and over. This was true for the global cognition measure, as well as all specific domains (other than executive functioning), with the strongest association being with the language domain. PRSs for other traits previously implicated in Alzheimer’s disease risk or cognition showed no evidence for association with cognition in any of our models.

In our GWAS analysis, we report two loci associated with change in the attention domain score; one associated with change in the first year of follow-up in an intergenic region on chromosome 21, and the second associated with change over the 1–5 year follow-up period near *EPAS1* on chromosome 2. Whilst *EPAS1* is the closest gene to the signal on chromosome 2, no further functional evidence was identified that implicates this gene. However, there was some evidence that this variant is associated with the expression of two more distal genes, *ATP6V1E2* and *CRIPT* in blood. However, colocalization evidence showed the causal evidence for the implication of these genes (in blood at least) to be weak. The protein encoded by *ATP6V1E2* is involved in proton-transporting ATPase activity in ubiquitous pathways such as *cellular response to stimuli*. *CRIPT* is the more interesting candidate at this locus, as the protein binds selectively to the third PDZ domain of the post-synaptic density protein 95 [44] and has a key role in learning and memory [45]. Whilst the lead variant at this locus has not previously been associated with any GWAS, this locus has previously been implicated in GWAS of brain morphology (rs17818315, *p* = 5 × 10^−8^) [46] and frontal lobe function (using the anti-saccade test, rs11125080, *p* = 2 × 10^−7^) [47]. No eQTL evidence provides support for the involvement of any gene at the intergenic chromosome 21 locus. However, chromatin interaction evidence does provide support for the role of *NCAM2* (>1.7 Mb away). This gene is involved in nervous system development and has been previously implicated in GWAS of educational attainment [30, 48].

Replication of these findings will be important to ensure they are robust effects, but this will be challenging given that these associations appear to be with specific aspects of change in the attention domain of cognition that may be hard to replicate, given the scarcity of studies of cognitively normal individuals of similar age, with such regular cognitive measures. Whilst the lack of replication amongst any of the previously reported cognitive change variants in our GWAS is notable, there are a number of reasons why this might be. The measured cognition phenotypes (and participants) differ subtly between studies, and it is possible that associations are with specific aspects of cognition (or in specific contexts, e.g. in Alzheimer’s disease patients or patients of the affective-to-psychotic spectrum), our current study may be under-powered to detect the effects given the relatively small sample size (*N* = 2515), or it is possible that the original findings were false positives. In Sherva et al. [11] their own replication analysis failed to find an association with their index variant, rs11023139 and reported replication only for a nearby variant, rs11606345 and Kamboh et al. [9] lacked a replication stage. It was not possible to look up the two associations that we reported in the previous relevant GWAS as they did not publish full summary statistics [9].

In contrast to the specific variant look-ups of previously reported cognitive change variants, we did show an association between the PRS for general cross-sectional cognitive ability (derived from Davies et al., GWAS [5]) and baseline global cognition (*p* = 5 × 10^−5^), as well as attention, learning, and language domains to a lesser extent (all *p* < 0.01). There was also some evidence that this PRS was associated with cognitive change in some models, e.g., slope 1 of attention domain (*p* = 0.002). This suggests that the lack of association observed between these individual previously published cognition variants and cognitive function or change in cognition in our study is likely due to a lack of power. Once those variants are combined into an allele score (which has greater statistical power than single variants), we identify the expected associations. This also highlights the importance of conducting adequately powered replication studies, although this remains challenging given the scarcity of large cohorts with both repeat measures of cognitive function and genotype data in non-clinical populations.

We found strong evidence for an association between an educational attainment PRS and the baseline measures of most cognitive scores, particularly with the language domain (*p* < 2 × 10^−16^). It is of note that the education PRS has an effect on cognitive measures so much later in the life course than the education phenotypes from which it was derived. The association with the education PRS may imply that there are shared genetic factors between education and later cognitive trajectories, and/or that there is a causal effect of education on later cognition as modelled here. This is consistent with the existing literature [49, 50] showing individuals with higher educational attainment maintain, on average, higher levels of cognitive function throughout life. We found no evidence for associations with all the other PRSs tested (Alzheimer’s disease, height, LDL cholesterol, physical activity, obesity, type 2 diabetes, systolic blood pressure, hearing loss, depression, smoking and alcohol consumption). This does not rule out associations that are small in magnitude, as our analyses were only sufficiently powered to identify large effects.

Other recent GWAS of cognitive function has applied methods such as genomic structural equation modelling to identify latent constructs of global cognition from several tests of specific domains. For example, de la Fuente et al. [51] estimate a *g* factor from seven cognitive tests and perform GWAS on that latent construct to identify variants associated with general cognitive function, as well as domain-specific function [51]. Those models have not yet been extended to examine change over time in latent constructs using, e.g. latent growth curve modelling and so their findings cannot be directly compared to ours, but work in this area is ongoing.

For a phenotype like cognition, change is an important and understudied trait in the context of GWAS. We have demonstrated the feasibility of conducting a GWAS of trajectories of repeated measures using GEE models. This approach allows for variant associations with specific features of the trajectories to be tested and provides a useful framework for future studies to investigate genetic risk factors of cognitive change.

Our study is limited by power, and we expect that it will be fruitful to conduct similar analyses in larger sample sizes with repeated cognitive measures (which are currently scarce). We have undertaken a large number of tests, which has further limited power and so replication (in independent studies) of specific findings that we report would be valuable to examine their robustness. Whilst the power of the PRS analysis could have been boosted using alternative methods that include variants below the genome-wide significance threshold [52–54], we chose not to do this for this study, as we wanted to limit the inclusion of pleiotropic variants (something that is not of concern when the aim is prediction). Whilst studies with heritable selection criteria can cause issues with collider bias, we have demonstrated here that selection on specific known genetic risk factors (and subsequent adjustment for these factors) is a tractable and robust study design in this context.

## Conclusion

We have conducted a GWAS of cognitive change, through GEE modelling of trajectories. Whilst the two genetic risk factors associated with cognitive change in later life in our analysis require replication, our demonstration of the feasibility of this approach should pave the way for larger future studies of this important question. The strong association that we observed between the education PRS, particularly in the language domain shows the impact of early life genetic predictors throughout the life course.

### Supplementary information

Supplementary Material

Supplementary Tables

## Data Availability

The datasets, including the redacted study protocol, redacted statistical analysis plan, and individual participants data supporting the results reported in this article, will be made available within 3 months from the initial request to researchers who provide a methodologically sound proposal. The data will be provided after its de-identification, in compliance with applicable privacy laws, data protection and requirements for consent and anonymization.

## Code availability

The code for the analyses is available upon request from the corresponding author.
