# Supplementary material for: Investigation of genetic determinants of cognitive change in later life
Source: Transl Psychiatry. 2024 Jan 18;14:31. doi: 10.1038/s41398-023-02726-6 (PMC10796929; doi:10.1038/s41398-023-02726-6)
Supplement: Supplementary file 1 — Supplementary Material [file 41398_2023_2726_MOESM1_ESM.docx]

**Description of Additional Supplementary Files**

File Name: Supplementary Table 1

Description: TOMMORROW neuropsychological battery

File Name: Supplementary Table 2

Description: Multilevel model results from the Global domain

File Name: Supplementary Table 3

Description: Sample characteristics

File Name: Supplementary Table 4

Description: Multilevel model results from the specific cognitive domains (attention, episodic, executive functioning, learning, and language)

File Name: Supplementary Table 5

Description: SNPs associated with specific domains and their association with all other domains.

File Name: Supplementary Table 6

Description: Look up of SNPs identified in (Davies et al., 2018) in our global cognitive function GWAS

File Name: Supplementary Table 7

Description: Look up of SNPs identified in (Hatoum et al., 2022) in our executive function GWAS.

File Name: Supplementary Table 8

Description: Look up of SNPs identified in (Mekki et al., 2022) in our language function GWAS.

File Name: Supplementary Table 9

Description: Association between cognitive functioning PRS (constructed from Davies et al., 2018) and all cognition models.

File Name: Supplementary Table 10

Description: Association between all risk factor PRSs and all cognition models.

**Supplementary Methods**

**TOMMORROW Trial – visit details**

There were two principal types of study visits in TOMMORROW: an in-clinic visit scheduled at 6-month intervals, and a comprehensive medical follow-up visit (CMFV) that is only required when a participant meets protocol‑specified trigger criteria that suggest potential cognitive decline. At the regular 6-month visit, health status and safety assessments included vital signs, weight, concurrent medications, medication compliance, adverse events, neurological exam, the Geriatric Depression Scale (GDS) (1), Columbia-Suicide Severity Rating Scale (2), and, on a yearly basis, physical exam, electrocardiogram, clinical chemistry, hematology, and urinalysis labs. Efficacy-related assessments included the TNB, MMSE, Alzheimer’s Disease Cooperative Study (ADCS) Clinical Global Impression of Change–MCI (CGIC–MCI) instrument (3), ADCS Prevention Instrument Project: Mail-in Cognitive Function Screening Instrument (ADCS MCFSI) (4), ADCS Activities of Daily Living–Prevention Instrument (ADL–PI) (5), Informant Questionnaire on Cognitive Decline in the Elderly (IQCODE) (6), and health outcomes measures. In-person participation was required at these regular visits, and while in-person project partner participation was strongly encouraged, it was not a requirement, as project partner data for these visits could be collected by phone if necessary.

**Computational processing time**

Parallel processing was used to complete ~9 million GEE models for each cognitive domain and global cognitive functioning (six outcomes). In total ~ 53 million models were completed. In order to manage the size of the data, data were subsetted into chromosomes. Each of the chromosome files were merged with the phenotype and covariate information. Along with submitting array jobs in Slurm that modelled each of the 22 chromosomes (ranging in size from 117,880 SNPs in chromosome 22 to 734,762 SNPs in chromosome 2), another level of parallelisation was included using the ‘doSNOW’ R package. Models were divided into arrays of 10,000 GEE models. Data were read in for those 10,000 which helps speed up computational time. 8.8 million GEE models took on average 5 hours to complete.

**Supplementary Figure 1.** **Panel A**. **TOMMORROW Trial study design**. TOMMORROW study design framework. (Note: The numbers of participants in each arm of the diagram reflect calculated participants needed for the prespecified statistical power, not the number actually enrolled in the study. After a reassessment by the sponsor in 2015, the drug effect size assumption was increased from 30% to 40%. This change resulted in a decrease in the number of required study participants to achieve study goals from the initial target of approximately 5800 to 2800 and reduced the number of efficacy endpoint events from 410 to 202.). Abbreviation: BRAA, biomarker risk assignment algorithm. **Panel B**. **Risk stratification scheme for the Biomarker risk assignment algorithm**. Abbreviations: APOE, apolipoprotein E; TOMM40, translocase of the outer mitochondrial membrane 40. **Panel C**. **TOMMORROW Trial enrolment procedure**. **Panel** **D**. **TOMMORROW study flow diagram.**


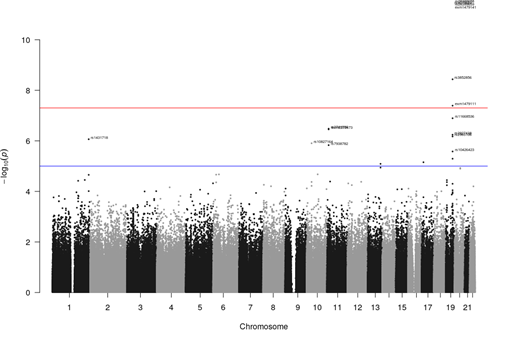

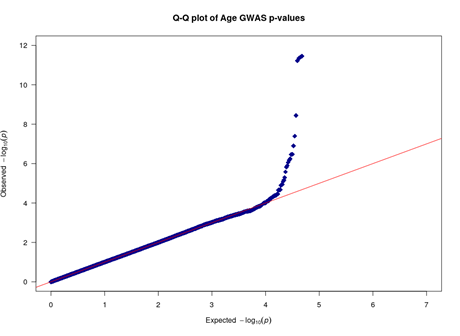


**Supplementary Figure 2A.** **Manhattan (left)/ quantile/quantile (QQ) plots (right) for age at baseline the TOMMORROW Trial**


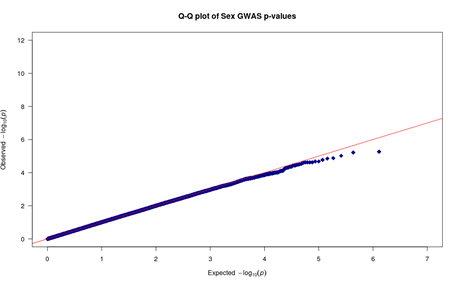

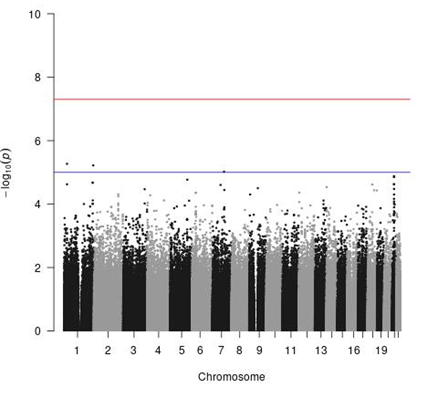


**Supplementary Figure 2B.** **Manhattan (left)/ quantile/quantile (QQ) plots (right) for sex the TOMMORROW Trial**

|  | Intercept | Baseline age | Slope 1 | Slope 2 |
| --- | --- | --- | --- | --- |
| Global | 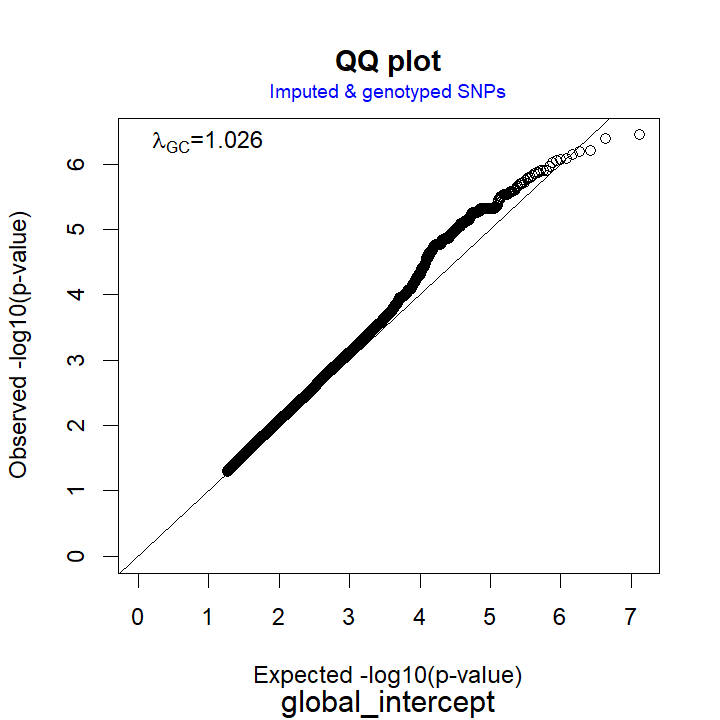 | 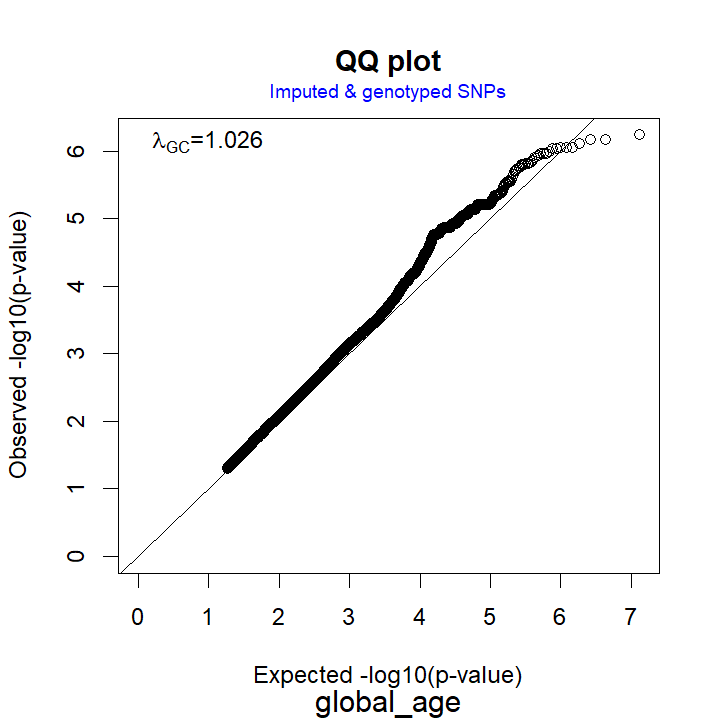 | 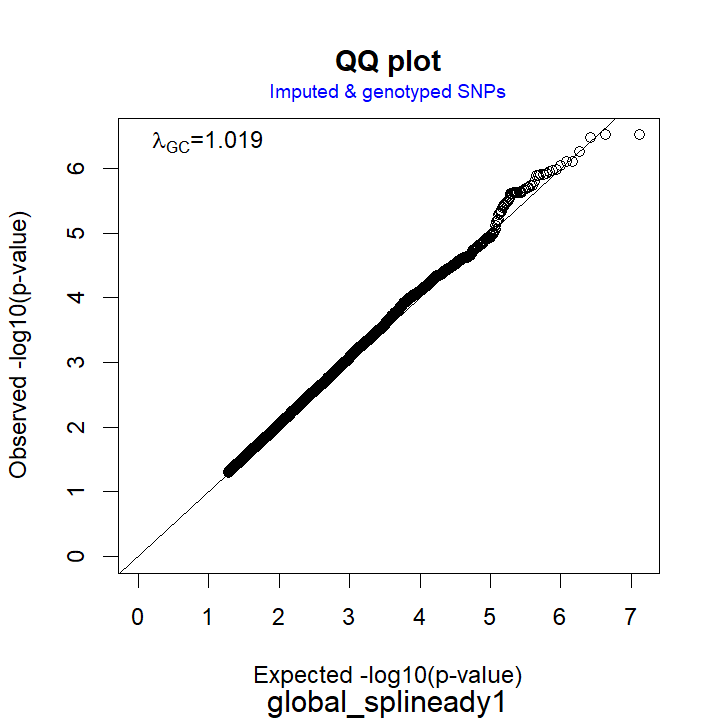 | 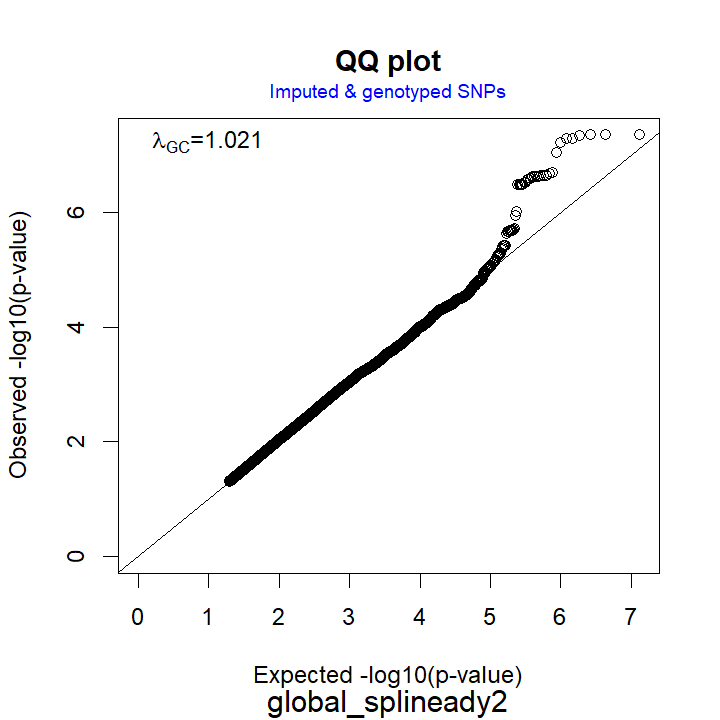 |
| Attention domain | 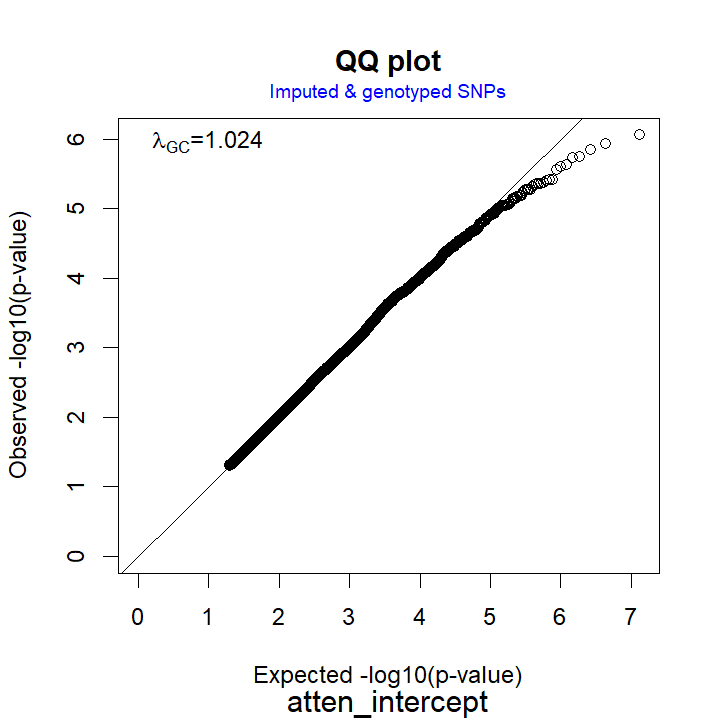 | 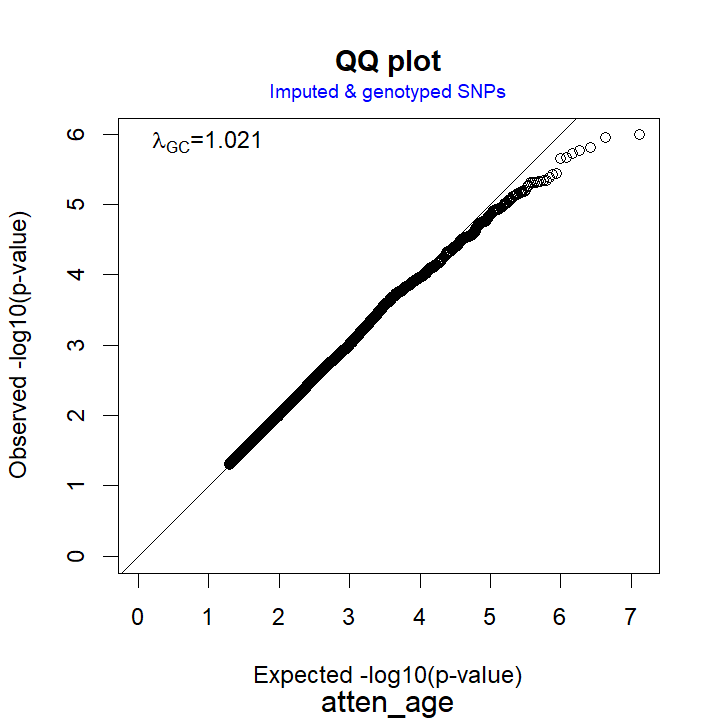 | 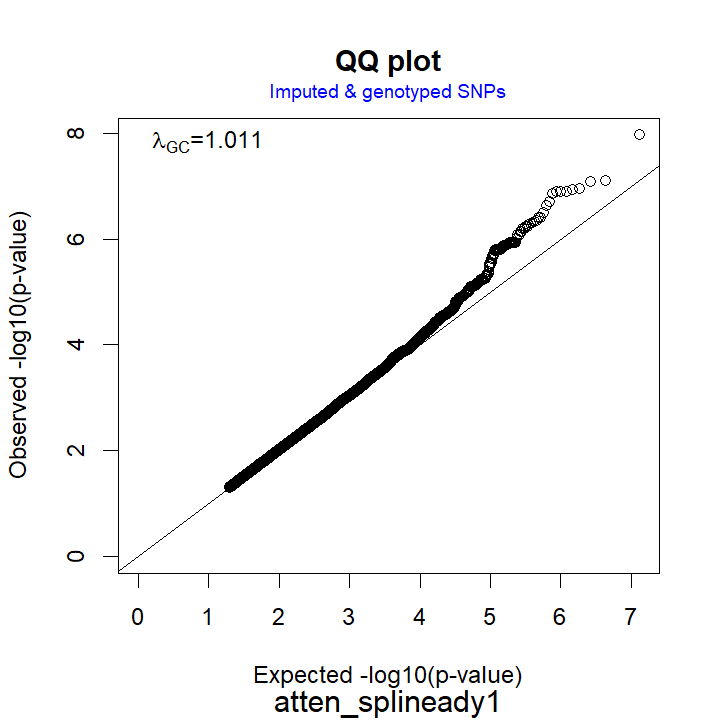 | 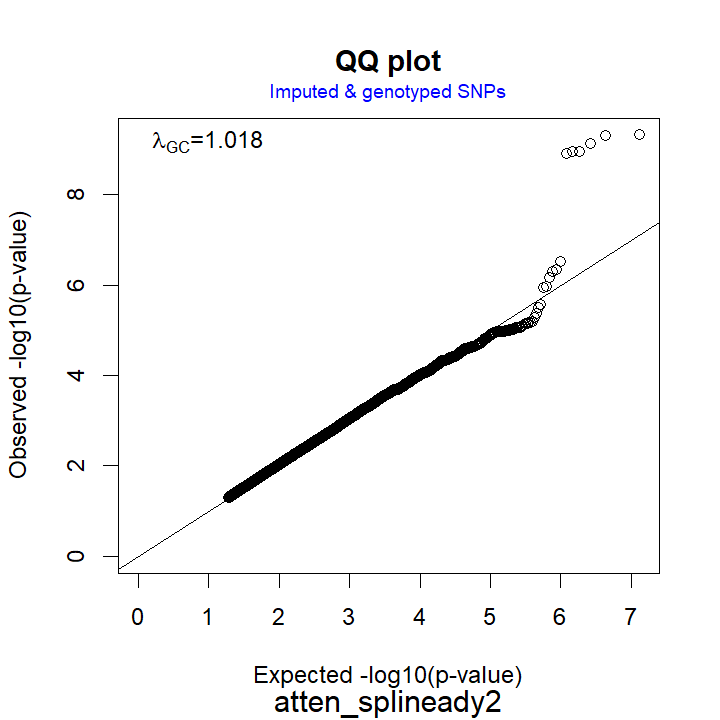 |
| Episodic domain | 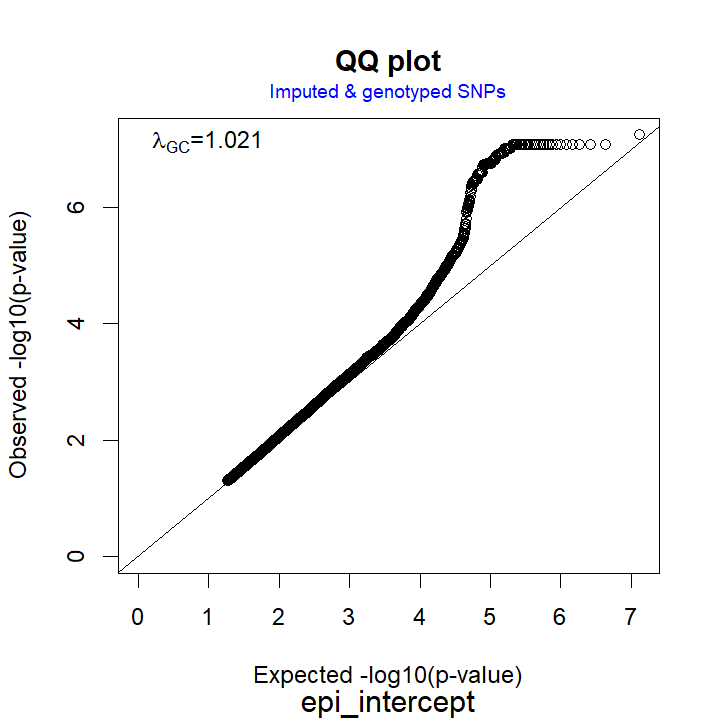 | 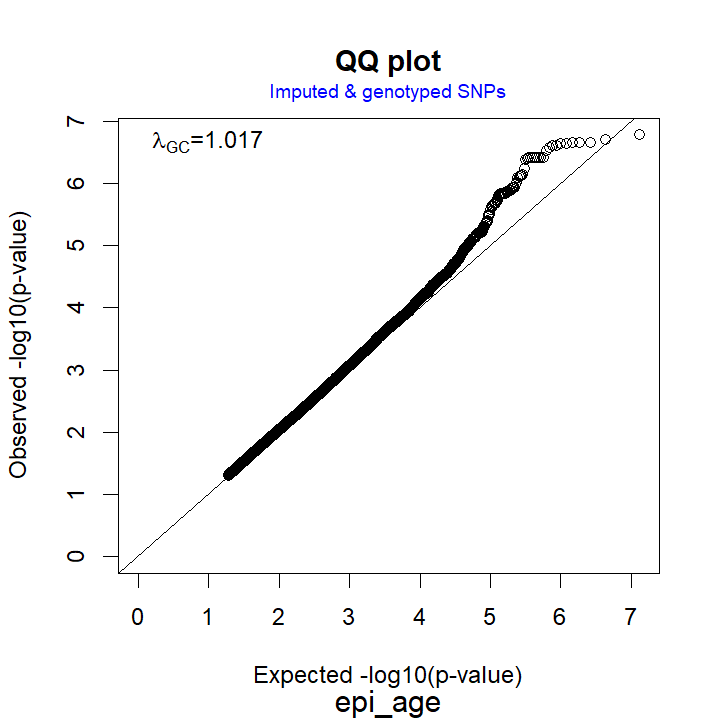 | 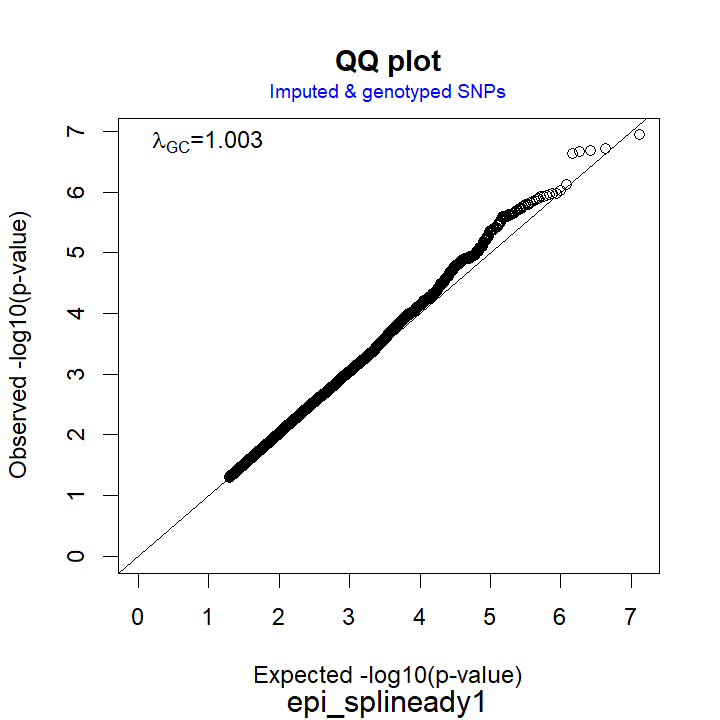 | 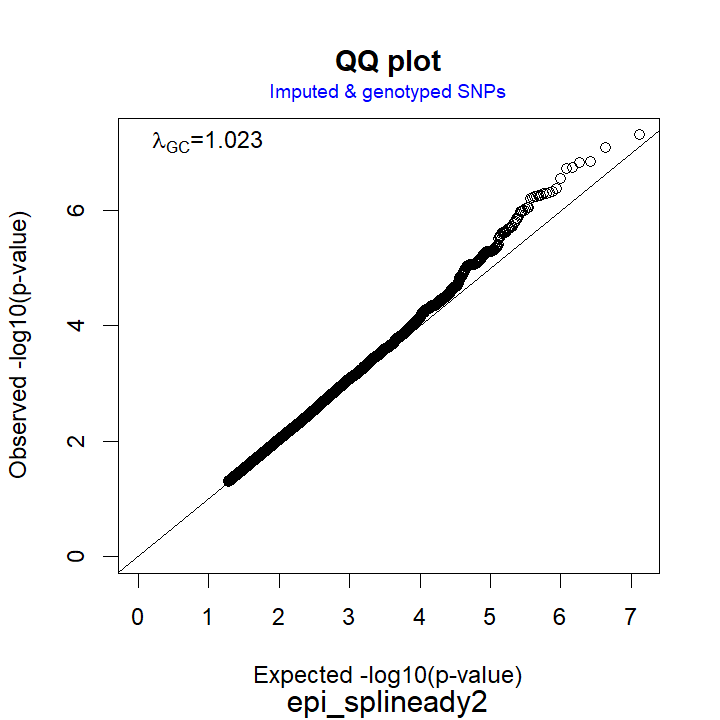 |
| Executive Function domain | 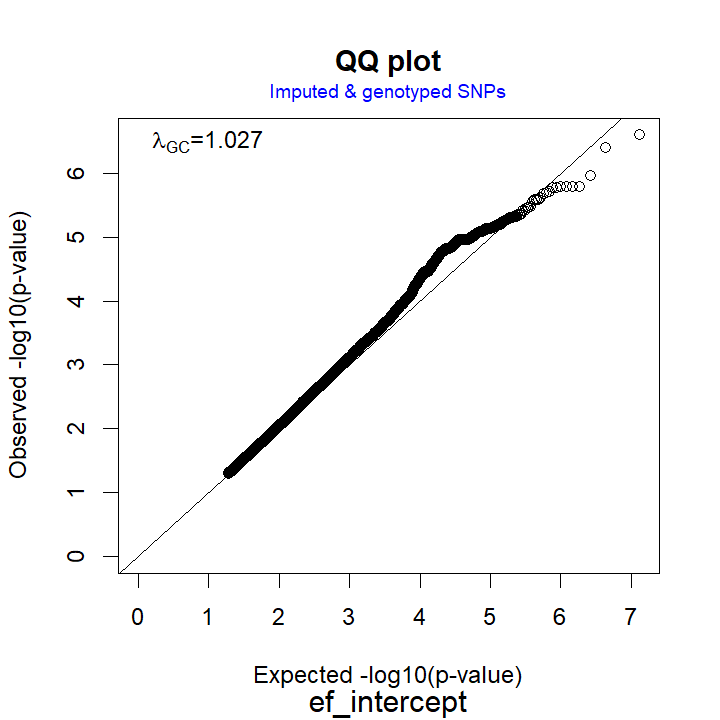 | 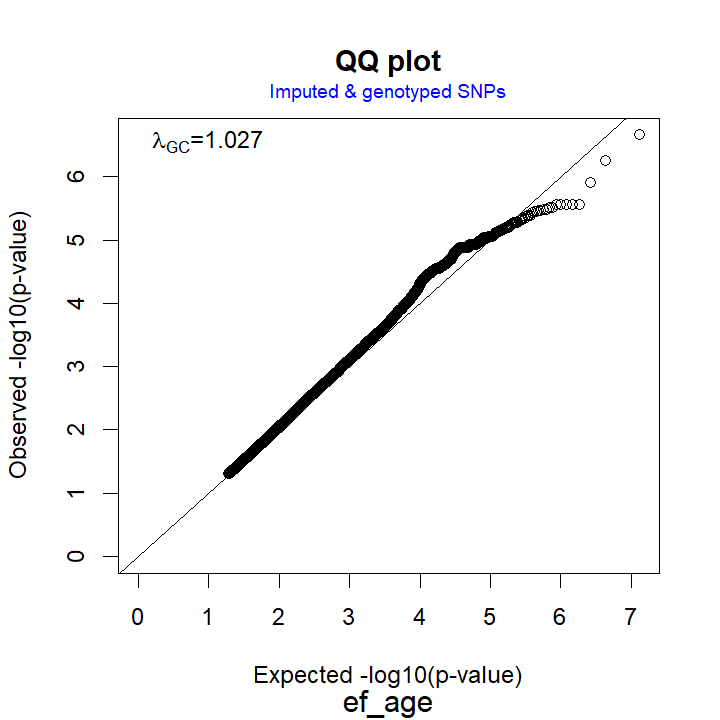 | 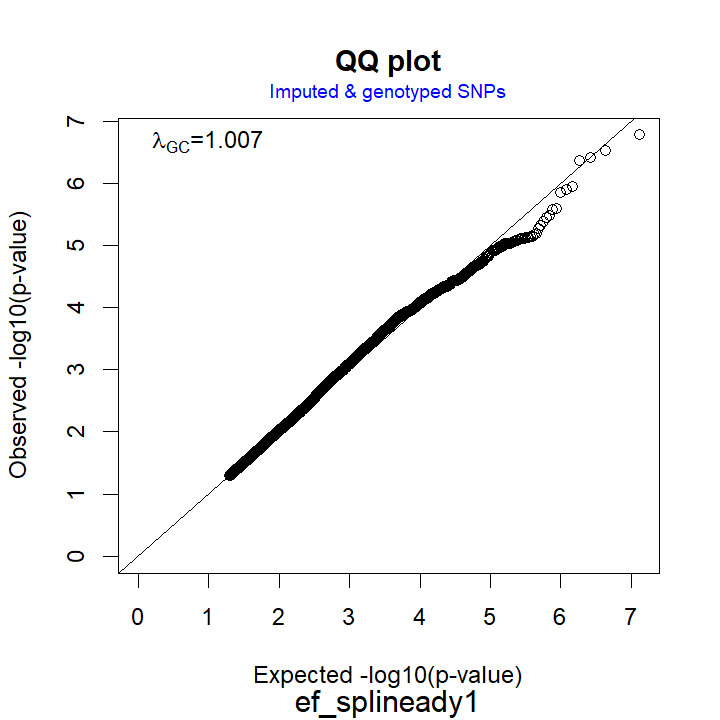 | 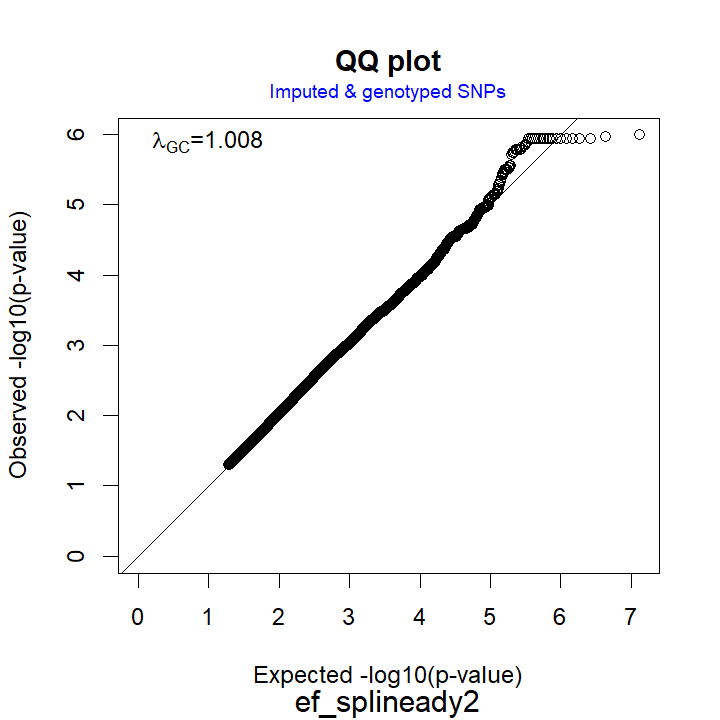 |
| Learning domain | 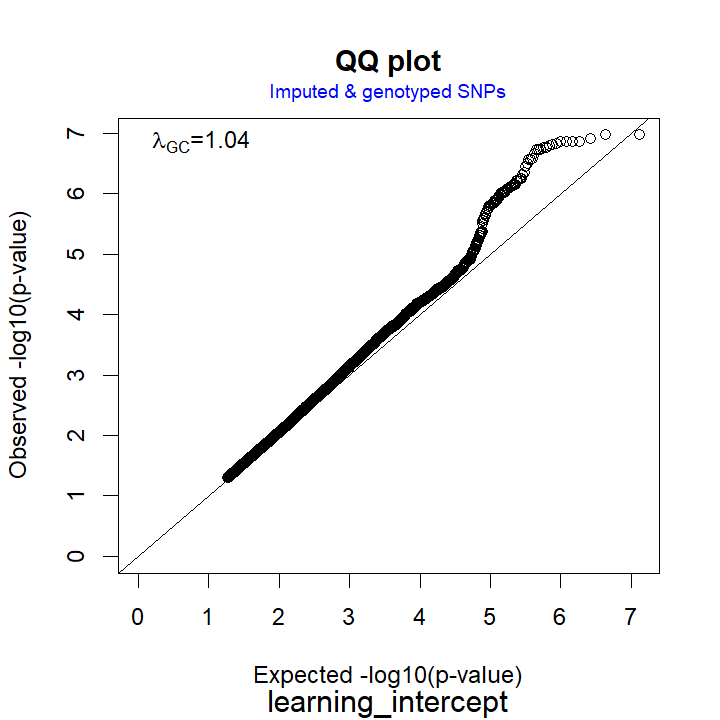 | 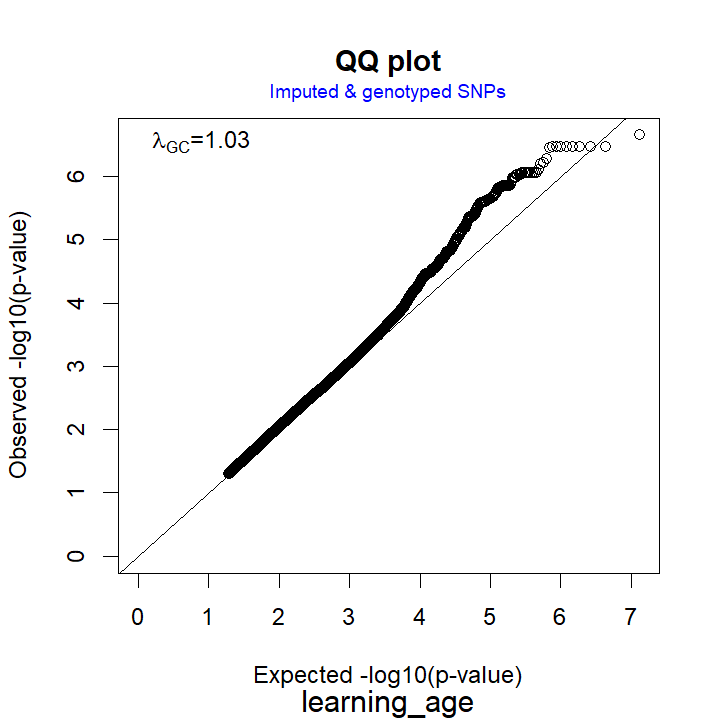 | 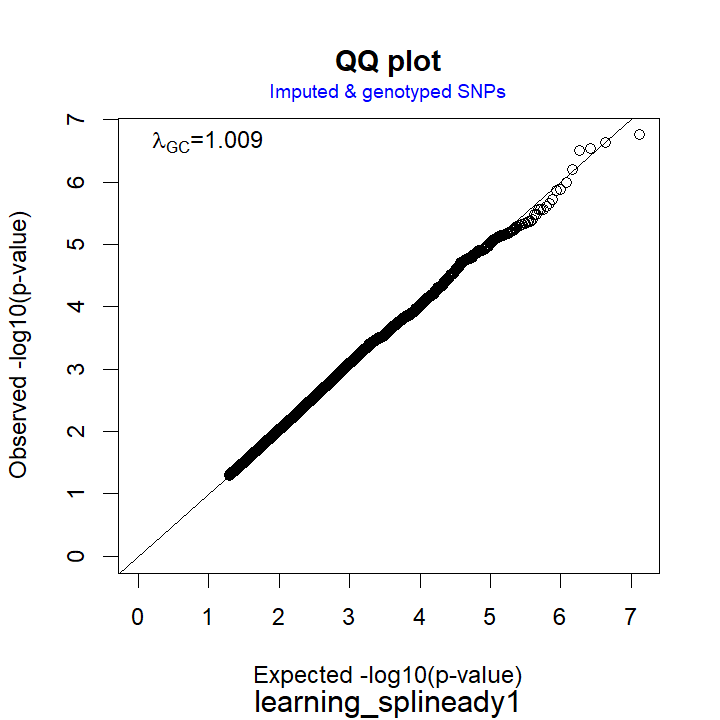 | 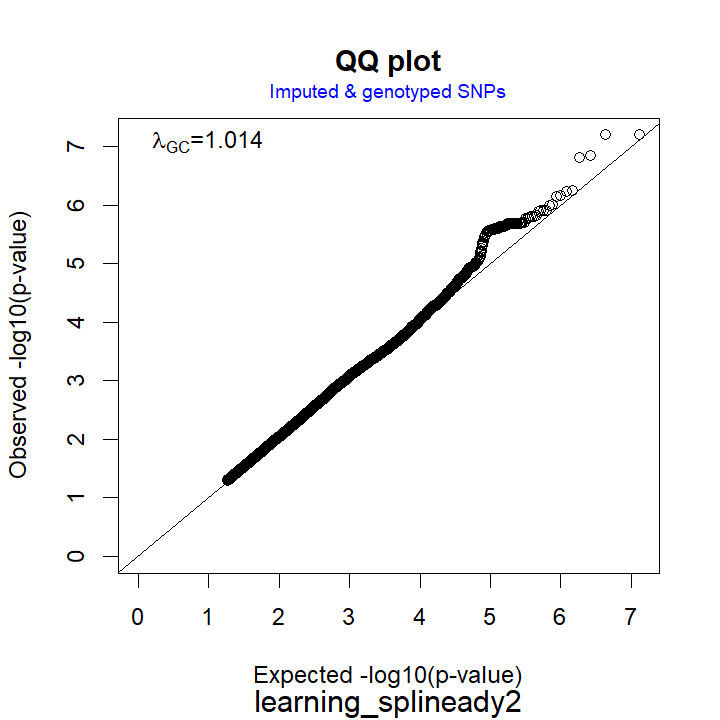 |
| Language domain | 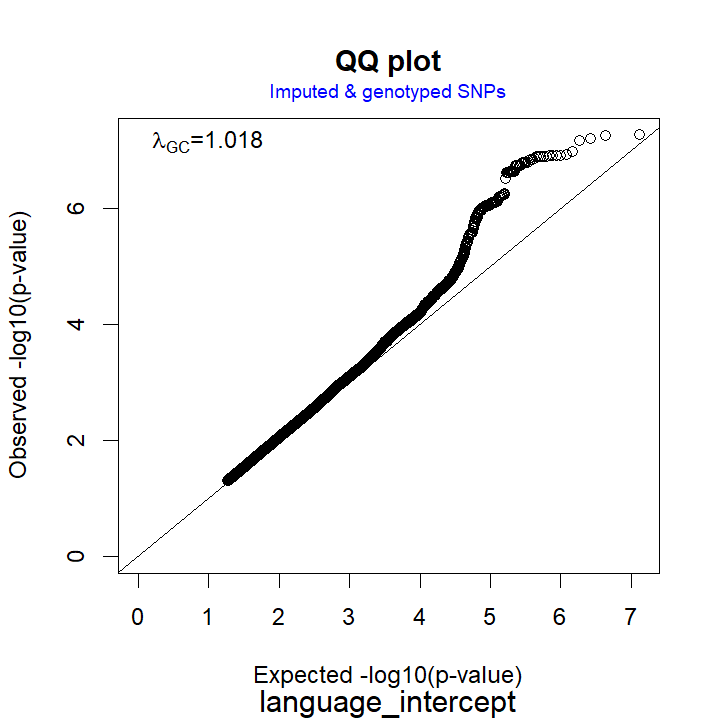 | 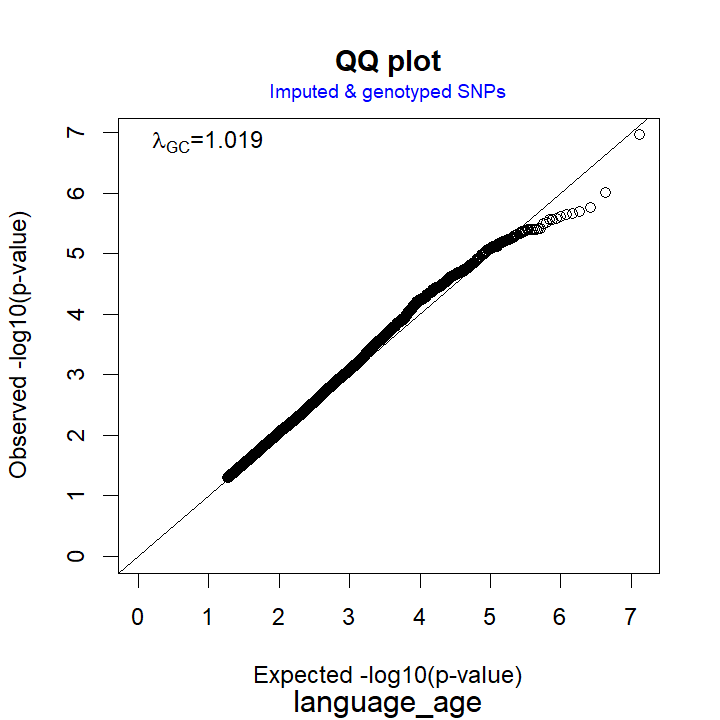 | 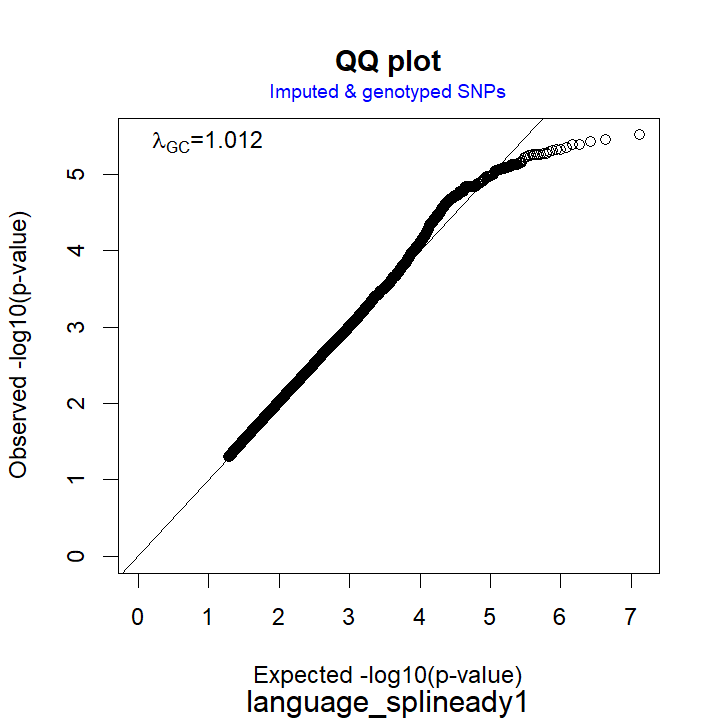 | 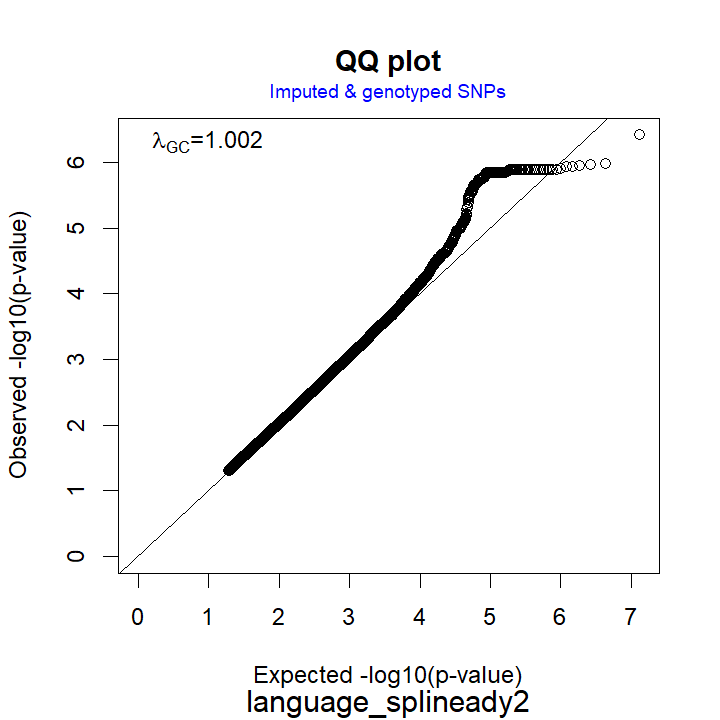 |

**Supplementary Figure 3. QQ plots of the 4 model parameters for all cognitive domains**

| Global | Intercept | Baseline age |
| --- | --- | --- |
|  | 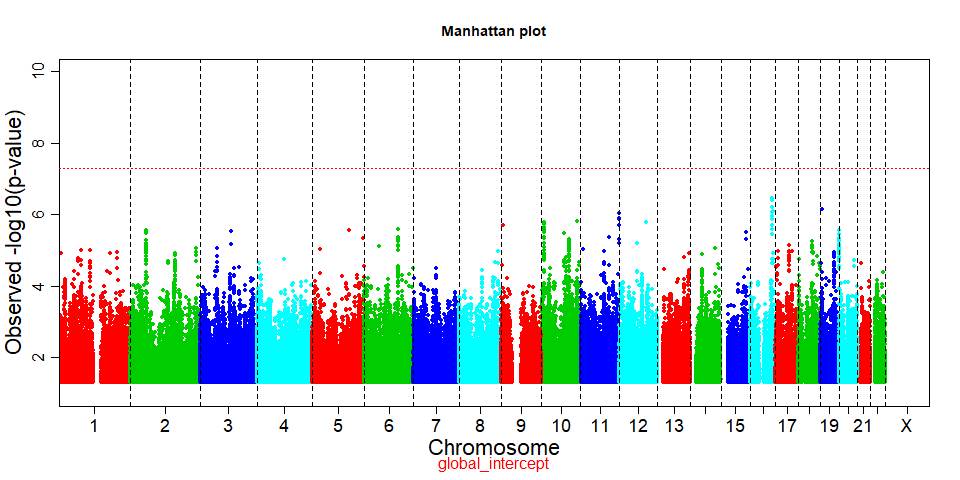 | 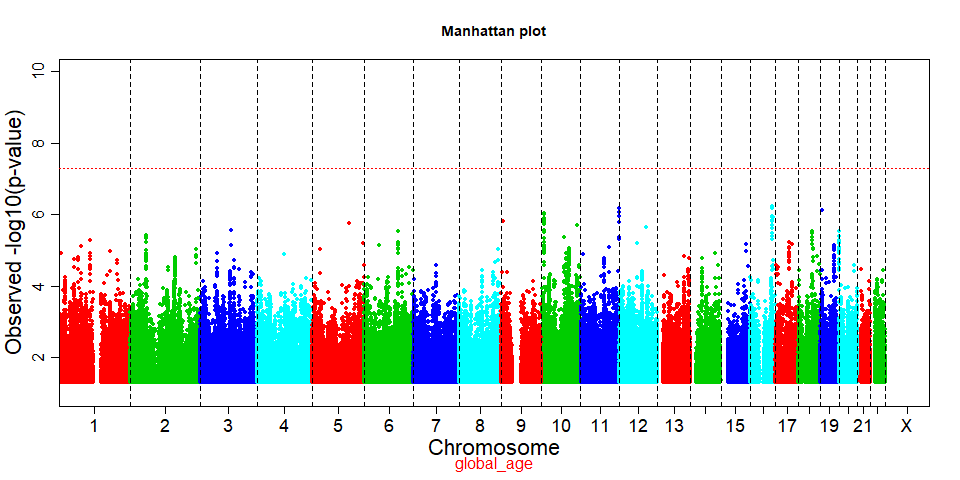 |
| Global | Slope 1 | Slope 2 |
|  | 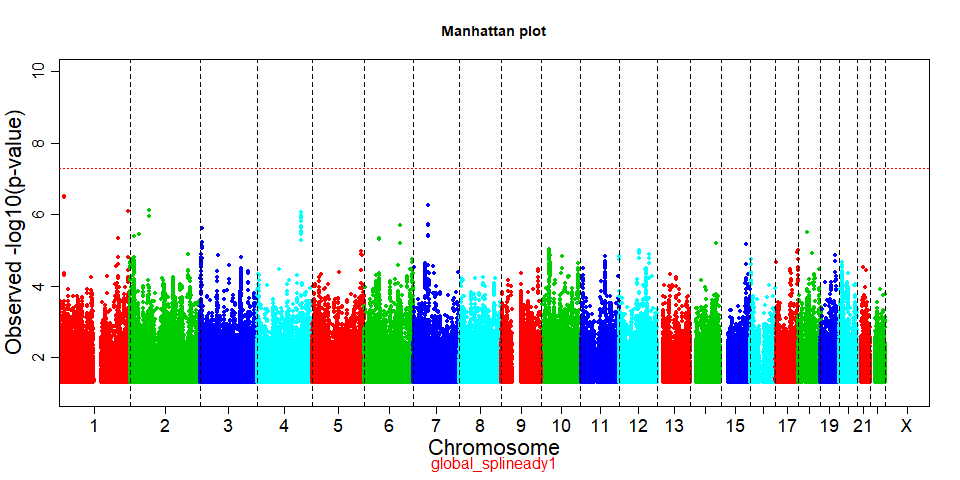 | 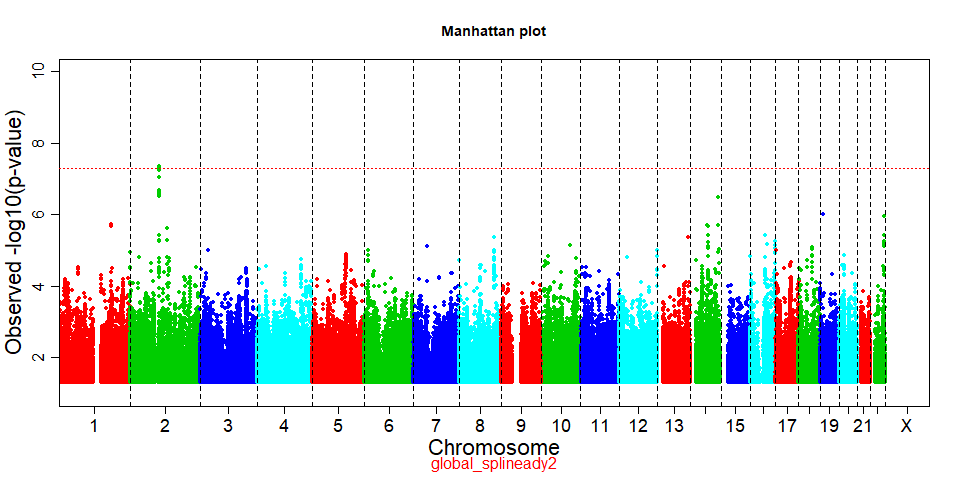 |
| Atten | Intercept | Baseline age |
|  | 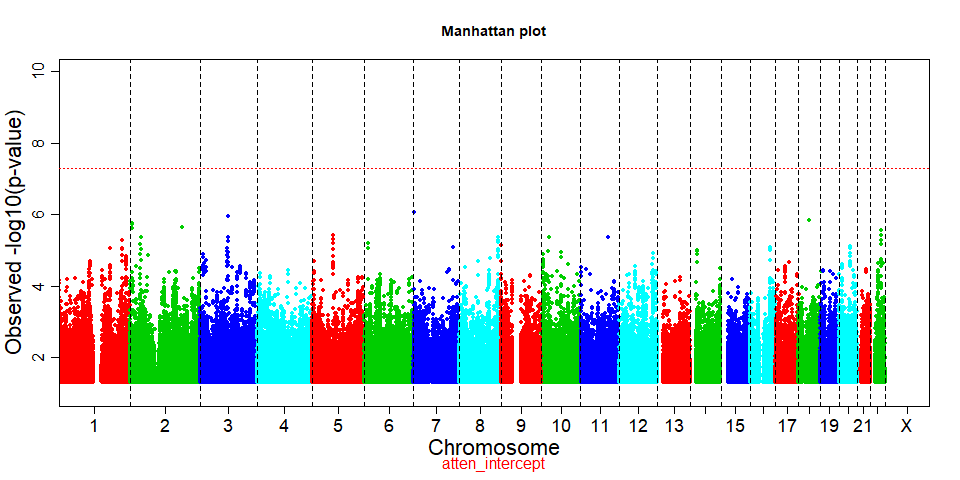 | 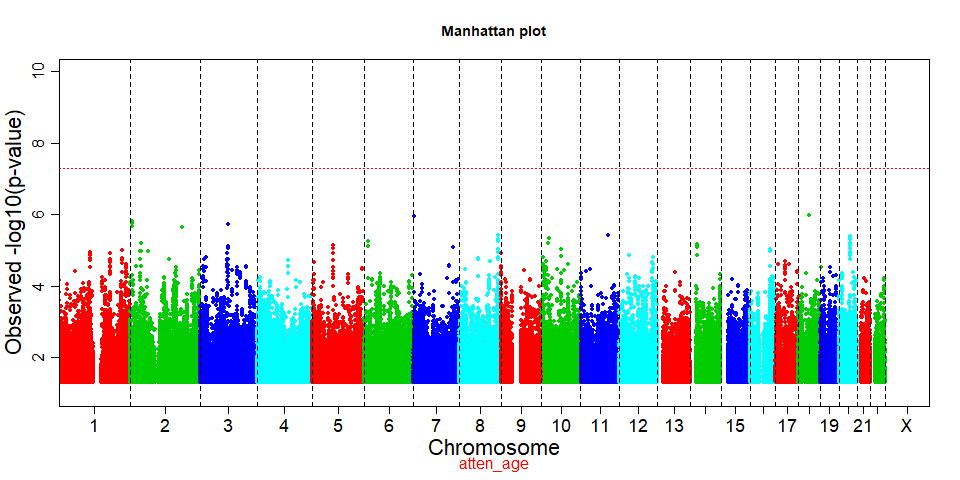 |
| Atten | Slope 1 | Slope 2 |
|  | 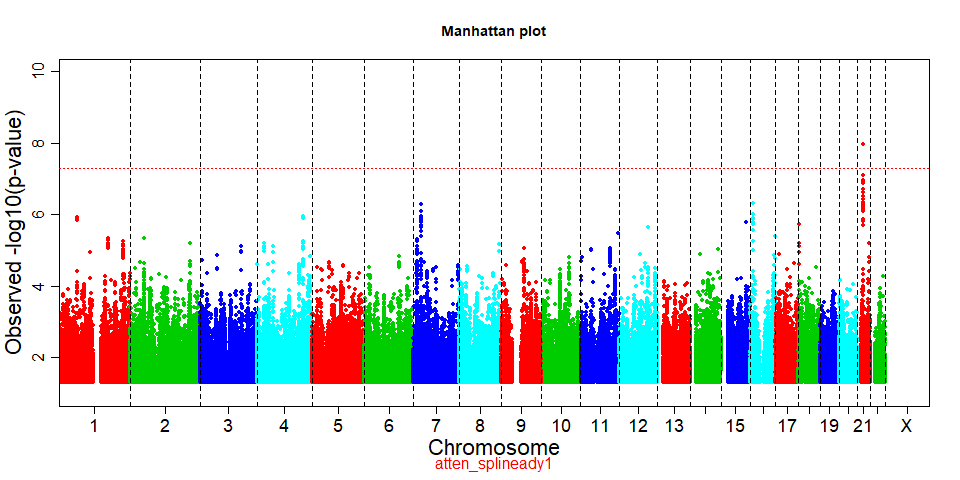 | 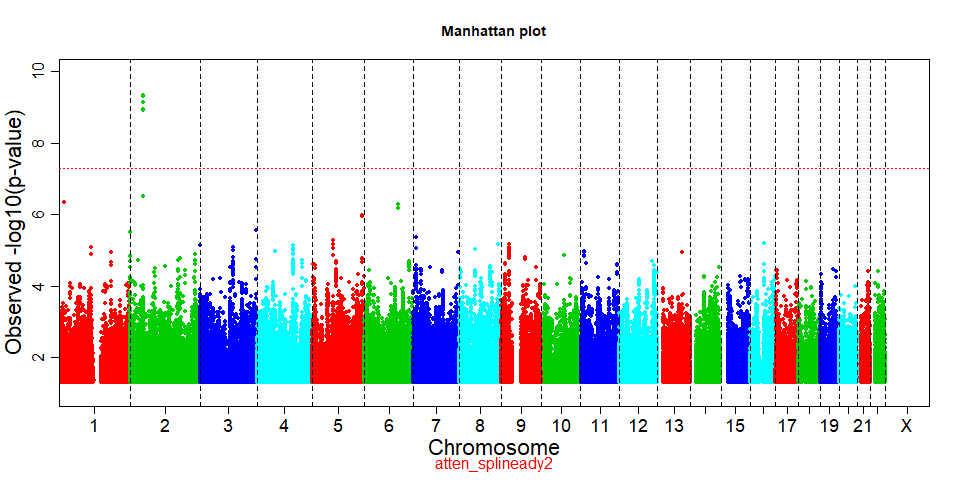 |
| Epi | Intercept | Baseline age |
|  | 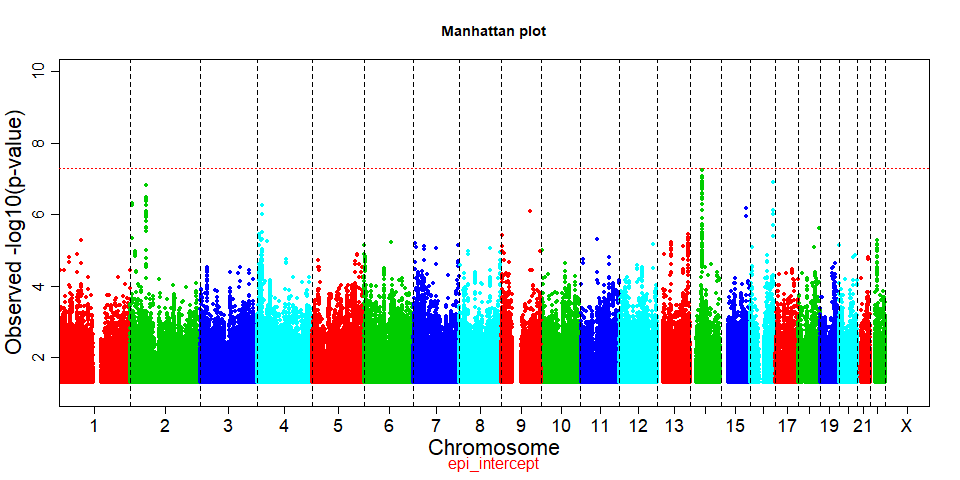 | 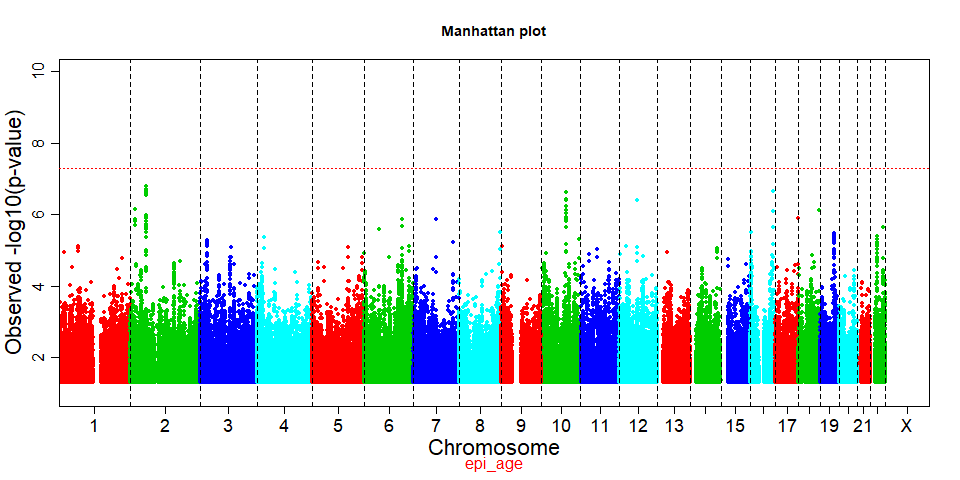 |

| Epi | Slope 1 | Slope 2 |
| --- | --- | --- |
|  | 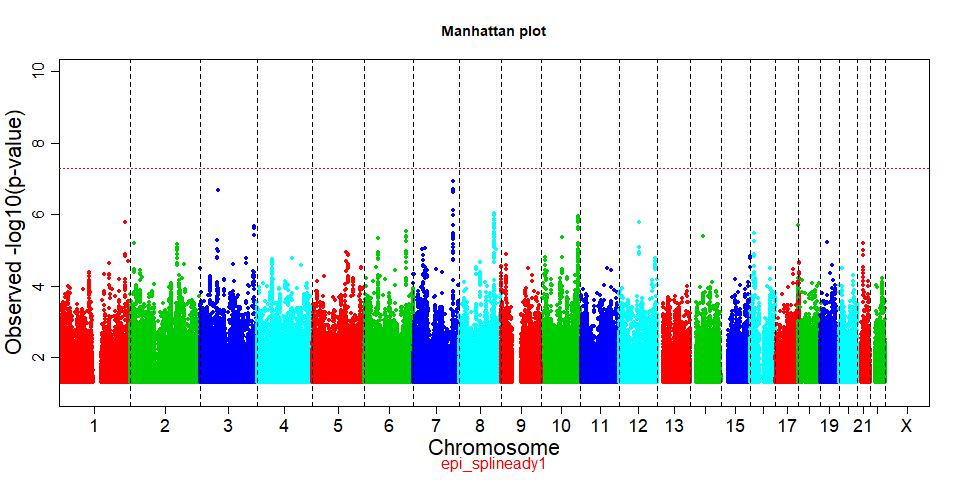 | 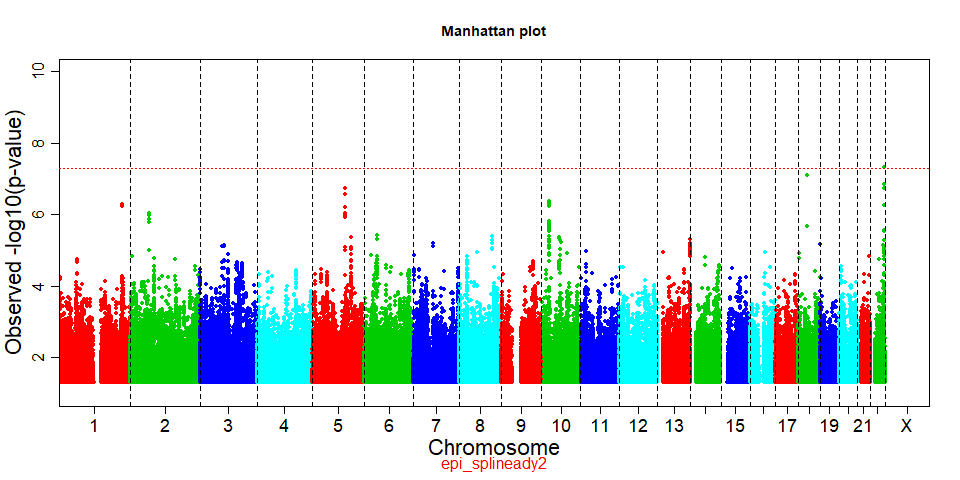 |
| Ex func | Intercept | Baseline age |
|  | 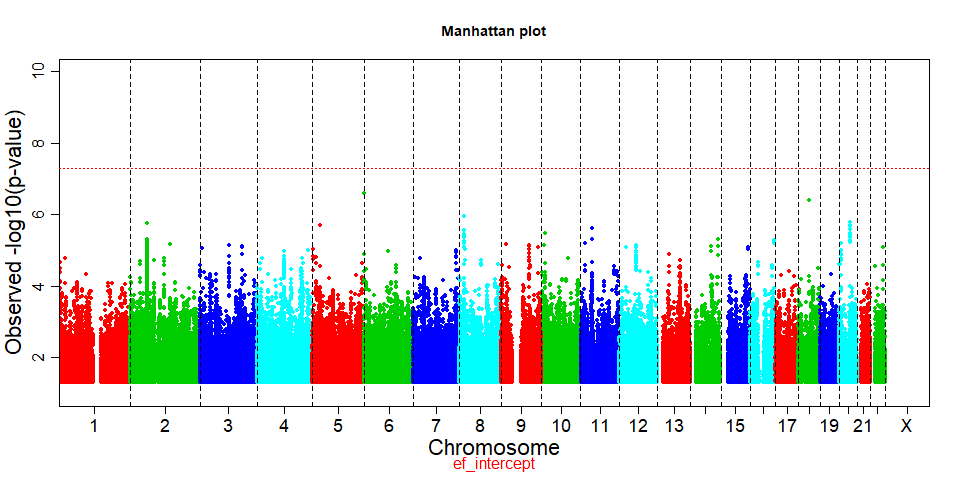 | 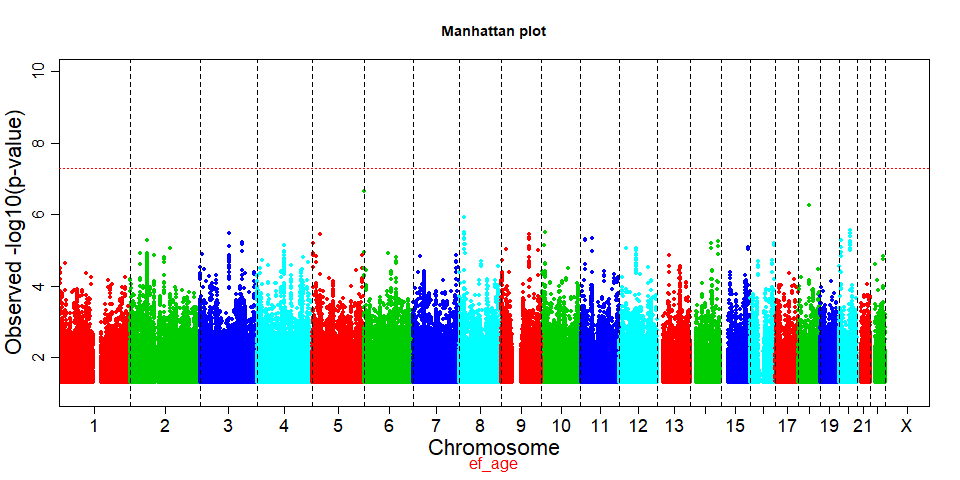 |
| Ex func | Slope 1 | Slope 2 |
|  | 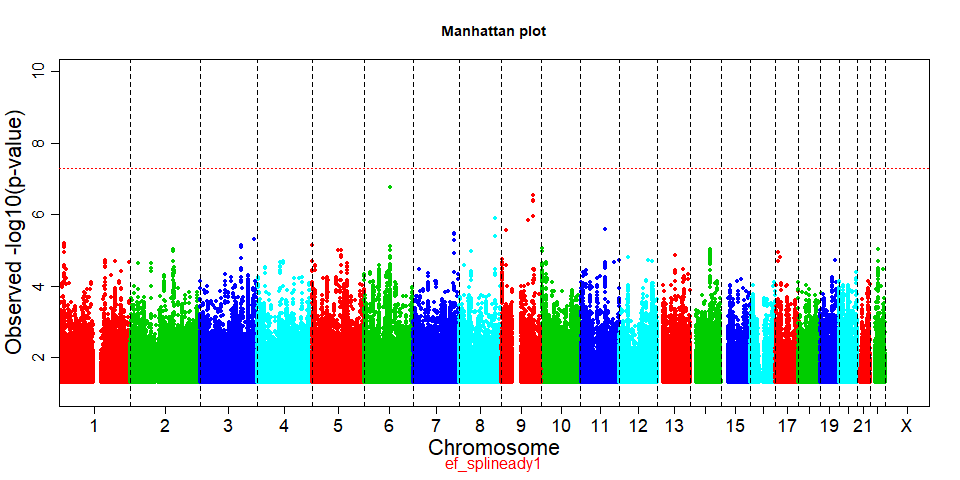 | 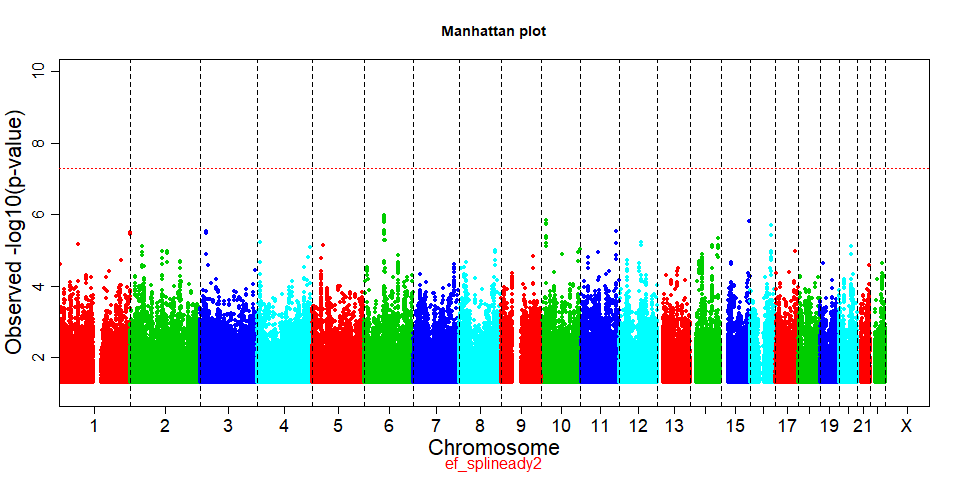 |
| Learn | Intercept | Baseline age |
|  | 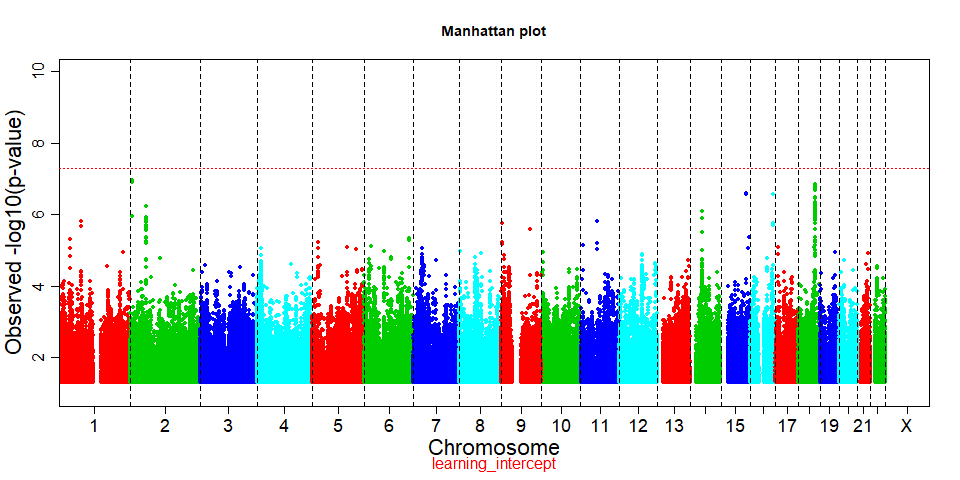 | 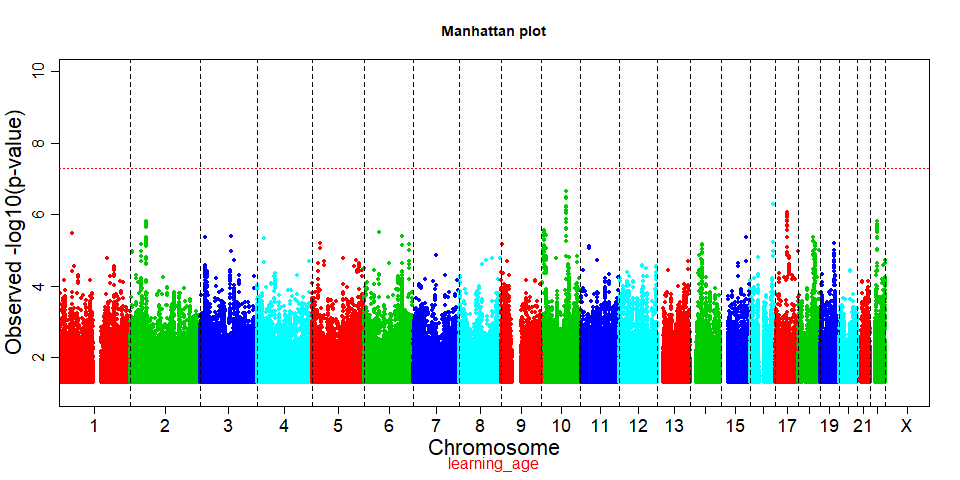 |
| Learn | Slope 1 | Slope 2 |
|  | 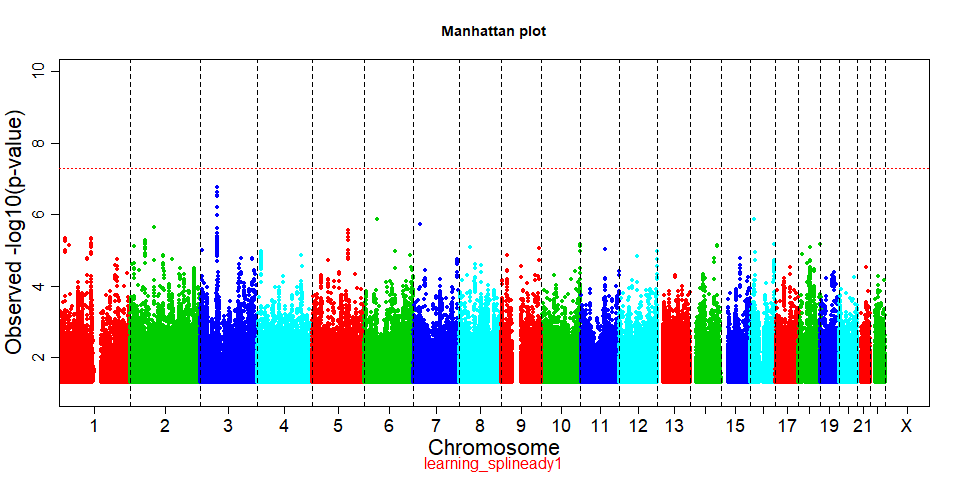 | 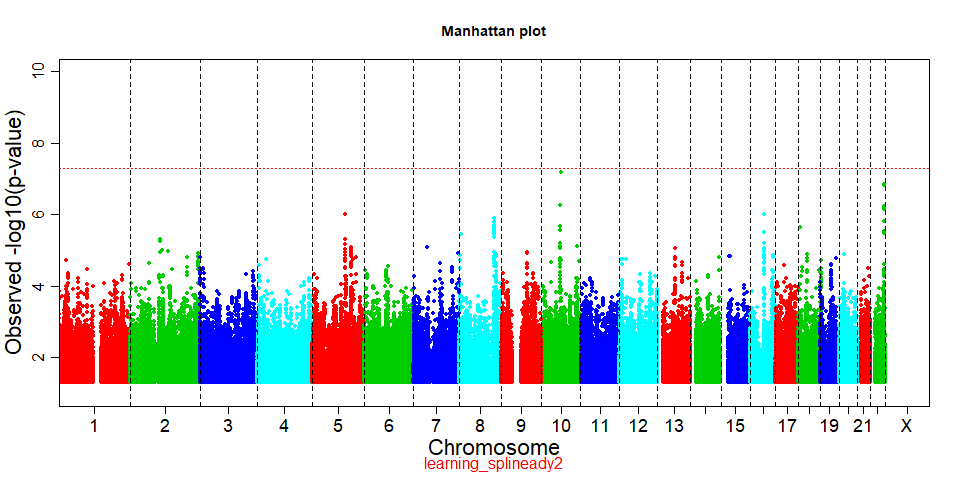 |

| Lang | Intercept | Baseline age |
| --- | --- | --- |
|  | 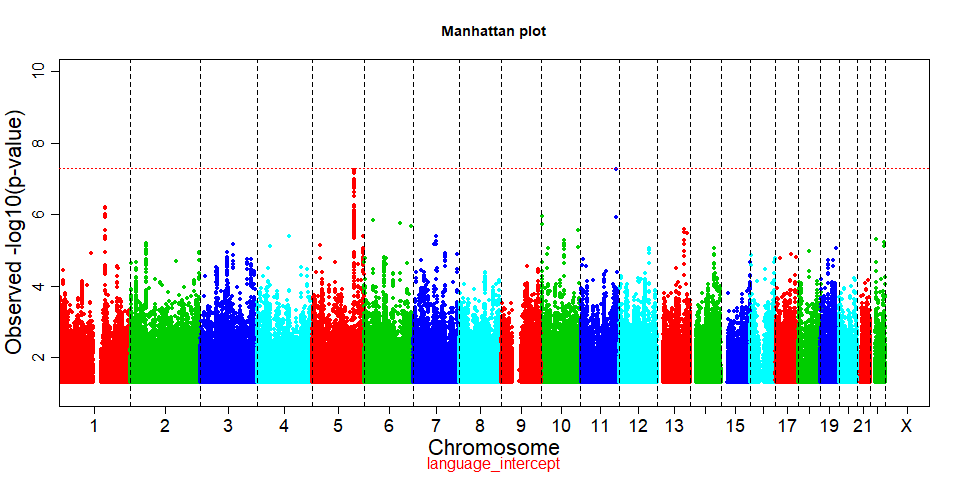 | 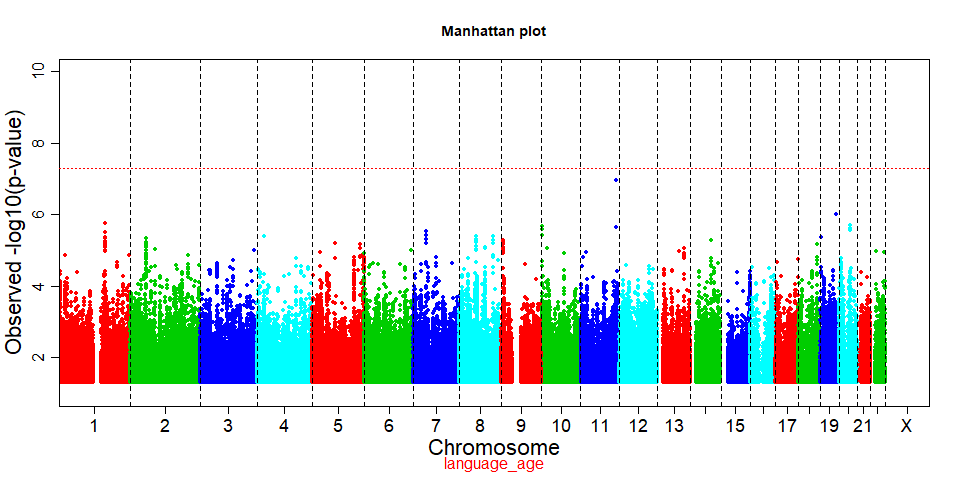 |
| Lang | Slope 1 | Slope 2 |
|  | 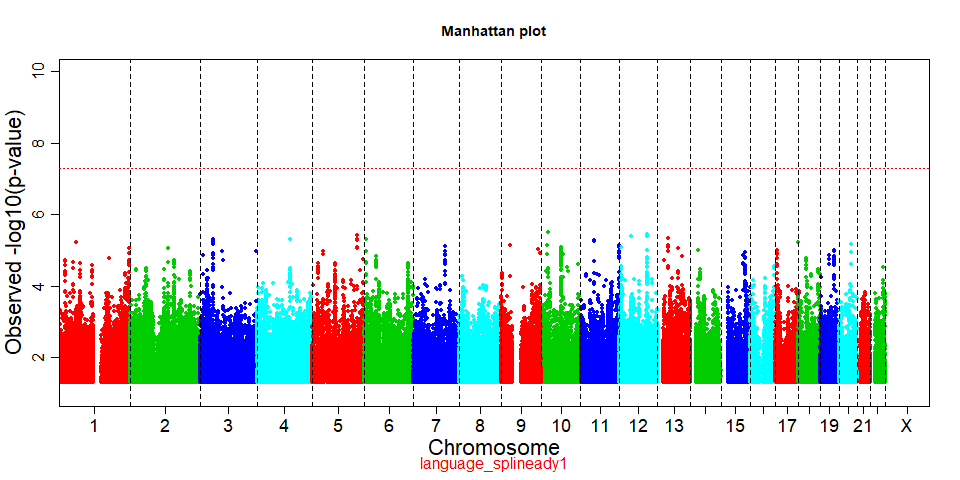 | 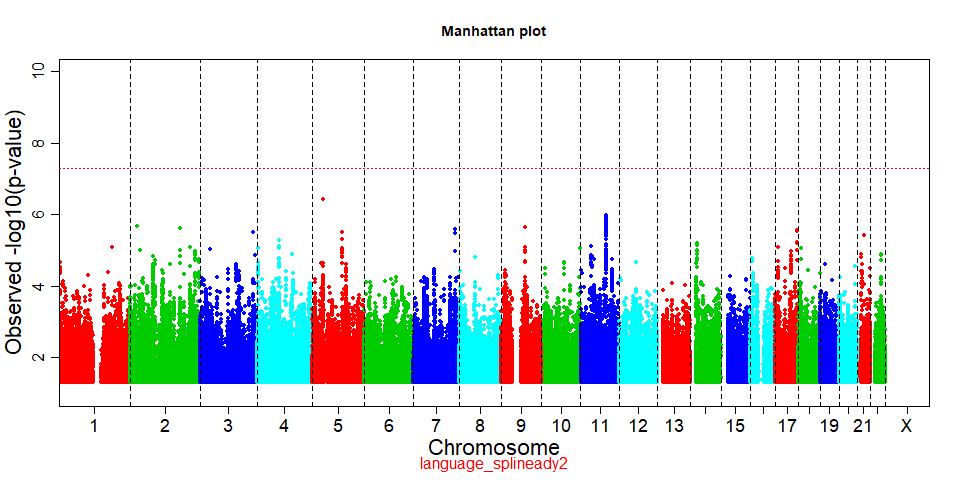 |

**Supplementary Figure 4. Manhattan plots of the 4 model parameters for all cognitive domains.**

**
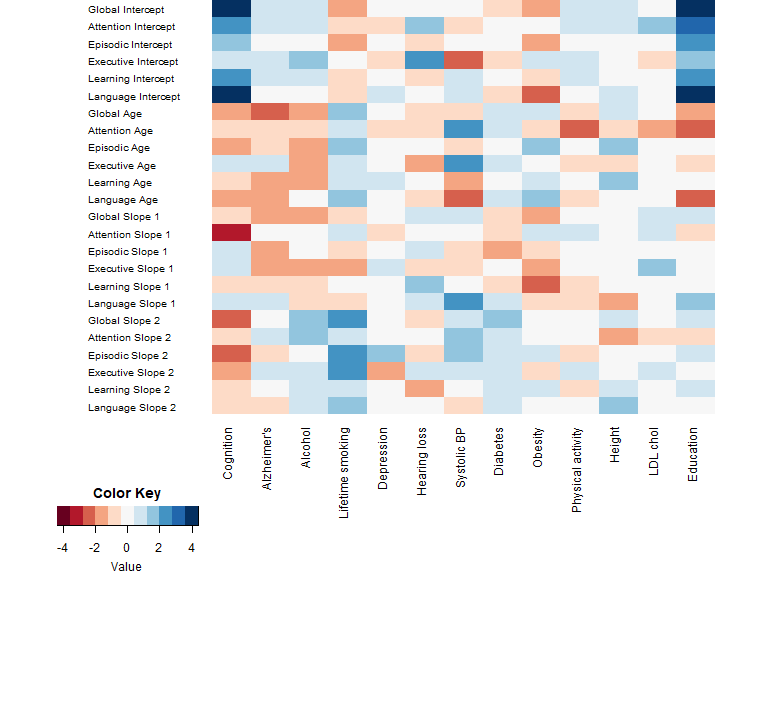
Supplementary Figure 5**. **Heatmap of Z-scores of the associations between polygenic risk scores (for the traits on the x-axis) and the cognitive trajectory parameters (4 parameters for each of the 6 domains, shown on the y-axis)**. Full results are displayed in Supplementary Tables 9 and 10.

**Supplementary Figure 6. Education PRS for the lowest, median and highest deciles for each cognitive outcome.** Age is set at the mean age, sex is set to female, TOMM40 and APOE are coded as low risk.

REFERENCES

1. Yesavage JA, Brink TL, Rose TL, Lum O, Huang V, Adey M, et al. Development and validation of a geriatric depression screening scale: a preliminary report. J Psychiatr Res. 1982;17(1):37-49.

2. Posner K, Brown GK, Stanley B, Brent DA, Yershova KV, Oquendo MA, et al. The Columbia-Suicide Severity Rating Scale: initial validity and internal consistency findings from three multisite studies with adolescents and adults. Am J Psychiatry. 2011;168(12):1266-77.

3. Schneider LS, Raman R, Schmitt FA, Doody RS, Insel P, Clark CM, et al. Characteristics and performance of a modified version of the ADCS-CGIC CIBIC+ for mild cognitive impairment clinical trials. Alzheimer Dis Assoc Disord. 2009;23(3):260-7.

4. Walsh SP, Raman R, Jones KB, Aisen PS. ADCS Prevention Instrument Project: the Mail-In Cognitive Function Screening Instrument (MCFSI). Alzheimer Dis Assoc Disord. 2006;20(4 Suppl 3):S170-8.

5. Galasko D, Bennett DA, Sano M, Marson D, Kaye J, Edland SD, et al. ADCS Prevention Instrument Project: assessment of instrumental activities of daily living for community-dwelling elderly individuals in dementia prevention clinical trials. Alzheimer Dis Assoc Disord. 2006;20(4 Suppl 3):S152-69.

6. Jorm AF, Jacomb PA. The Informant Questionnaire on Cognitive Decline in the Elderly (IQCODE): socio-demographic correlates, reliability, validity and some norms. Psychol Med. 1989;19(4):1015-22.
